# Utilizing a divalent metal ion transporter to control biogenic nanoparticle synthesis

**DOI:** 10.1093/jimb/kuad020

**Published:** 2023-08-16

**Authors:** Manasi Subhash Gangan, Kyle L Naughton, James Q Boedicker

**Affiliations:** Department of Physics and Astronomy, University of Southern California, Los Angeles, CA 90089, USA; Department of Physics and Astronomy, University of Southern California, Los Angeles, CA 90089, USA; Department of Physics and Astronomy, University of Southern California, Los Angeles, CA 90089, USA

**Keywords:** Synthetic biology, Biosynthesis, Nanoparticles, Cadmium sulfide, Metal ion transport

## Abstract

Biogenic synthesis of inorganic nanomaterials has been demonstrated for both wild and engineered bacterial strains. In many systems the nucleation and growth of nanomaterials is poorly controlled and requires concentrations of heavy metals toxic to living cells. Here, we utilized the tools of synthetic biology to engineer a strain of *Escherichia coli* capable of synthesizing cadmium sulfide nanoparticles from low concentrations of reactants with control over the location of synthesis. Informed by simulations of bacterially-assisted nanoparticle synthesis, we created a strain of *E. coli* expressing a broad-spectrum divalent metal transporter, ZupT, and a synthetic CdS nucleating peptide. Expression of ZupT in the outer membrane and placement of the nucleating peptide in the periplasm focused synthesis within the periplasmic space and enabled sufficient nucleation and growth of nanoparticles at sub-toxic levels of the reactants. This strain synthesized internal CdS quantum dot nanoparticles with spherical morphology and an average diameter of approximately 3.3 nm.

**One-Sentence Summary:**

Expression of a metal ion transporter regulates synthesis of cadmium sulfide nanoparticles in bacteria.

## Graphical Abstract

**Graphical Abstract:**
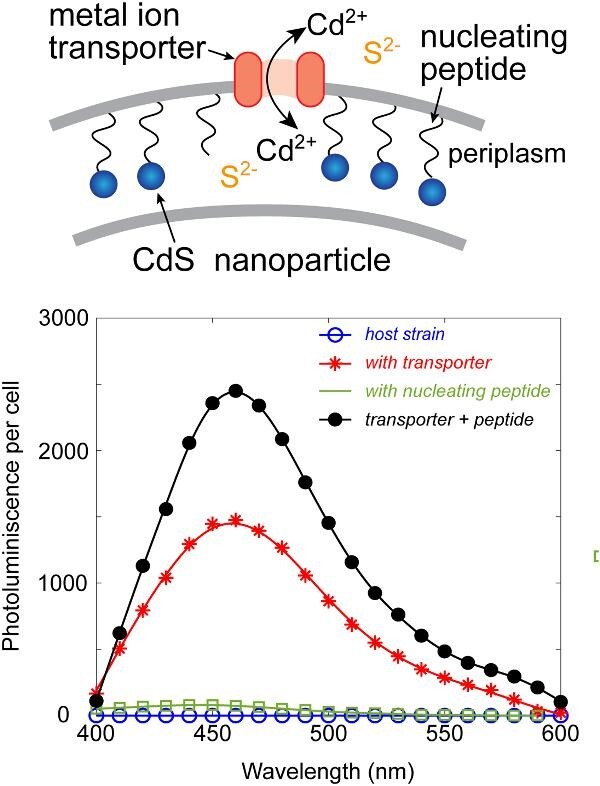
Coupled expression of metal transporter and nucleating peptide facilitates biosynthesis of CdS nanoparticles in *E. coli* cells.

## Introduction

Synthesis of nanoparticles by living organisms has recently garnered attention owing to the natural ability of many bacterial species to interact with and manipulate metals and semimetals (Boedicker et al., 2021; Chellamuthu et al., 2019; Choi & Lee, 2020; Klaus et al., 1999; McFarlane et al., 2015; Wakatsuki, 1995; Zou et al., 2021). Several bacterial species have evolved specialized biochemical pathways to perform redox reactions with heavy metals, which is associated with cellular respiration and reducing the toxicity of these elements (Challagulla et al., 2020; Mahle et al., 2020; Lloyd, 2003; Pikuta et al., 2007). Bacteria are known to naturally accumulate transition metals into nanostructures that assist in various cellular functions (Cai et al., 2007; Yan et al., 2015). Bacteria thus make an attractive system to facilitate the biosynthesis of nanoparticles by providing alternative to traditional chemical synthesis (Ghosh et al., 2021).

Over the years, microbial species have been isolated and shown to synthesize nanoparticles derived from different metals either intracellularly or extracellularly (Akid et al., 2008; Hussain et al., 2016; Naik et al., 2002; Wu & Ng, 2017; Yang et al., 2022). As an extension to this work, many research groups have actively employed synthetic biology to control and improve biosynthesize of nanoparticles (Chen et al., 2009; Chen et al., 2014; Mao et al., 2003). These approaches have improved yield and have enabled the control of nanoparticle properties such as size, shape, and composition (Bai et al., 2009; Dunleavy et al., 2016; Dahoumane et al., 2017; Kumar et al., 2010; Mao et al., 2003; Narayanan & Sakthivel, 2010, Singh et al., 2011). Previous attempts have successfully modified biochemical pathways derived from different species of microorganisms for cost-effective and eco-friendly synthesis of nanoparticles (Huston et al., 2021). Among the various efforts to synthesize nanoparticles biologically, there is growing interest directed towards biosynthesis of cadmium sulfide (CdS) nanoparticles (Bai et al., 2009; Chen et al., 2009; Gupta et al., 2021; Tripathi et al., 2014), due to the interesting electronic and photochemical properties of this materials that has applications in optics, optoelectronics, biology, and medicine (Klaus-Joerger et al., 2001; Mandal et al., 2006). Limitations of current approaches include poor control over nanoparticle properties and the use of precursor materials at concentrations toxic to living cells.

In the present study, we developed a strategy to overcome these hurdles and synthesize CdS nanoparticles at low concentrations of cadmium with control over the location of synthesis. We leverage a previously reported simulation to predict how cellular properties influence nanoparticle synthesis (Naughton & Boedicker, 2021). Informed by the model, we engineered a cell to express a broad-spectrum metal importer, ZupT, and a metal nucleating peptide to synthesize CdS nanoparticles in the periplasmic space of *Escherichia coli*. Increased cadmium ion uptake mediated by ZupT was predicted to increase the concentration of metal ions in the periplasmic space, where the presence of nucleating peptides sequesters these ions to initiate formation of cadmium sulfide nanoparticles. With our genetic module, we successfully biosynthesized CdS nanoparticles of size 3.3 nm, as validated by SEM and X-ray diffraction. The constructs demonstrated here as well as the general approach of simulation-guided engineering of bacterial cells for biogenic nanoparticle synthesis should prove useful to further advance microbial technologies for the synthesis of nanomaterials.

## Materials and Methods

### Plasmid and Strain Construction

All the plasmids and primers used in these studies are listed in Tables S1 and S2. Synthetic constructs expressing outer membrane ZupT and native OmpA tagged with nucleating peptide were derived from pBAD24 (Guzman et al., 1995) and pDSG372 (Glass & Riedel-Kruse, 2018), respectively. New plasmids were constructed via Gibson assembly by following the protocol from New England Laboratories. Chemically competent DH5α (NEB TOP10) cells were used as a host during plasmid construction and *E. coli ΔzupT* (CGSC #10 305) was used as the host strain for experimental measurements. Sequences of constructed plasmids were verified via Sanger sequencing.

To construct *pzupT_OM_* plasmid, *zupT* ORF was amplified from genomic DNA of *E. coli* MG1655 and introduced downstream of arabinose inducible promoter *P_BAD_* on pBAD24 (Fig. S1A). Plasmid was further modified, by tagging the 5′ end of *zupT* ORF with a DNA sequence encoding a signal peptide derived from *ompA* gene (Nangola et al., 2010; Thie et al., 2008), as shown in Fig. S1B. This tag would traffic the ZupT protein to the outer membrane.

*pompA_np* plasmid was assembled in three steps. *ompA* ORF amplified from *E. coli* MG1655 genomic DNA was inserted downstream to P_lacIq_. A linker peptide, Gly_4_Ser_4_, was added to the C-terminus of *ompA* ORF, as can be seen in Fig. S1C. Next the sequence encoding a peptide known to bind CdS surfaces was added (Peelle et al., 2005). Multiple peptide sequences have been shown to nucleation CdS nanoparticle formation (Flynn et al., 2003; Mao et al., 2003; Wang et al., 2022). The production of CdS nanoparticles was quantified for *E. coli ΔzupT* + *pzupT_OM_* cells expressing four of these peptides, see Fig. S2. In this work, we selected peptide sequence ‘EEGGHHHGGEE’ (data shown with filled black circles corresponding to the strain *ΔzupT* + *ompA_np4*), as cells expressing this peptide resulted in cell extract with the highest photoluminescence.

### Growth Conditions

*Escherichia coli* strains were grown in Luria-Bertani (LB) broth at 37°C and 200 rpm for all experiments. Strains were stored at −80°C in glycerol stocks. Strains were revived via overnight culture and washed thrice with 1X PBS prior to inoculating secondary cultures.

### Chemicals

Arabinose was purchased from Sigma-Aldrich and was dissolved in distilled water at 20% (w/v) concentration to prepare stock solution. 1 M solution of zinc chloride was prepared in distilled water and used to test zinc uptake in *E. coli* cells expressing ZupT in outer membrane. Stock solutions of 10 µg/ml cadmium chloride in 10 mM Tris-Cl (pH 7.0) and 0.25 M sodium sulfide (both from Sigma-Aldrich) in distilled water. All stock solutions were stored at 4°C.

### Isolation of Outer Membrane

To isolate outer membrane, the protocol described by Park et al., 2015 was used (Park et al., 2015). In brief, *E. coli ΔzupT* cells transformed with *pzupT_OM_* plasmid were induced with arabinose and harvested 2 hrs after induction. Cells were lysed using 10 mg/ml of lysozyme at room temperature, followed by DNase treatment (1 mg/ml DNase in 100 mM PMSF) on ice for 30 mins. Cells were pelleted down at 6000 rpm for 10 mins. Supernatant obtained in this step was further centrifuged at 20 000 rpm for 10 mins. Outer membrane (OM) particles were then washed with 1% Tween 20 and suspended in 100 µl of 1X PBS. The suspension was used to run 10% SDS gel for the detection of ZupT band at approximately 26 kDa position.

### Quantification of Zinc Uptake

A volume of 25 ml secondary cultures of all the strains were grown in LB broth until optical density (OD) −0.2. Cultures were then induced with 0.01, 0.1, and 1% arabinose and incubated for another 2 hrs, after which cells were suspended in 1 mM of zinc chloride solution for 1 hr. Cells were then harvested and analyzed for intracellular zinc uptake using a commercial zinc assay (QuantiChrom™ Zinc Assay Kit, BioAssay Systems).

### Growth Assay

Secondary cultures of *E. coli ΔzupT*, and *E. coli ΔzupT* transformed with (a) *pzupT_OM_* or with (b) *pompA_np* or with (c) both *pzupT_OM_* and *pompA_np* were inoculated in fresh 5 ml of liquid LB medium containing sodium sulfide at 1% inoculum. Cultures were incubated for 2 hrs before inducing them with 1% arabinose, which was then followed with incubation for another 2 hrs. At the end of the incubation, cells were pelleted down and transferred to sterile 10 mM Tris-Cl containing cadmium chloride and incubated further for 2 hrs. Note that the incubation was carried out at 37°C under shaking conditions at 200 rpm. We tested three combinations of sodium sulfide and cadmium chloride for the bacterial growth, as follows: (1) 0 µg/ml cadmium chloride and 0 mM sodium sulfide; (2) 1 µg/ml cadmium chloride and 0.0025 mM sodium sulfide; and (3) 10 µg/ml of cadmium chloride and 0.25 mM sodium sulfide. Growth was measured in terms of colony forming units (CFU) per ml by spotting 5 µl of cultures withdrawn at every hour on LB plated selected for appropriate antibiotic(s). The similarity between the calculated doubling time of *E. coli ΔzupT* and other three cultures was confirmed with Student's *t*-test (GraphPad).

### Biosynthesis of CdS Nanomaterials

In parallel experiments, 25 ml secondary cultures were started for *E. coli ΔzupT* and *E. coli ΔzupT* transformed either with *pzupT_OM_* or with *pompA_np* or with *pzupT_OM_* and *pompA_np* at 1% inoculum in LB supplemented with a variable concentration of sodium sulfide. Cultures were grown to 0.2 OD and incubated an additional 2 hrs with 1% arabinose induction. Cells were then washed thrice with 10 mM Tris-Cl (pH 7.0) and suspended in a variable concentration of cadmium chloride solution prepared in 10 mM Tris-Cl (pH 7.0) for an additional 1 hr of incubation. At this point, cell density of the culture was measured at 600 nm before isolation of cells by centrifugation and resuspension in 1 ml of distilled water. To isolate intracellular content, cell membrane was disintegrated by heating bacterial suspension at 100°C for 30 mins. The resultant solution was centrifuged at 13 000 rpm for 30 mins to collect the supernatant. A volume of 200 µl of supernatant was used for measurement of photoluminescence and absorbance values, which were normalized with respect to the OD values of the cultures to obtain photoluminescence as well as absorbance spectra per cell.

### Analysis of Photoluminescence

A volume of 200 µl of supernatant was excited at 365 nm and emission values were recorded at wavelengths ranging from 400 to 600 nm with 10 nm of interval using a plate reader (TECAN infinite M200PRO).

### Determination of Cadmium Sulfide Nanoparticles Concentration in the Supernatant

Spectroscopic method described by Yu et al., 2003 (Yu et al., 2003) was used to determine the concentration of cadmium sulfide nanoparticles in supernatant isolated from bacterial cells. A volume of 500 µl supernatant was measured for the absorbance at wavelengths from 350 to 500 nm using spectrophotometer (SPECTRONIC 200, Thermo Scientific) at room temperature. Wavelength (λ) at which first excitonic absorption peak was observed was used to calculate the size (D) of the particle using following equation:


(1) \begin{eqnarray*}
D &=& \left( { - 6.6521 \, {\mathrm{*}}\, {{10}}^{ - 8}} \right){\lambda }^3 + \left( {1.9557 \,{\mathrm{*}} \,{{10}}^{ - 4}} \right){\lambda }^2\\
&& - \left( {9.2352\, {\mathrm{*}}\, {{10}}^{ - 2}} \right)\lambda + \left( {13.29} \right)\end{eqnarray*}

Values obtained for nanoparticle size were then plugged in Equation (2) to calculate molecular extinction coefficient (ϵ) of supernatant.


(2) \begin{equation*}\epsilon = 21536{\left( D \right)}^{2.3}\end{equation*}

Molecular extinction coefficient was in turn used to calculate concentration of cadmium sulfide nanoparticles in supernatant using Beer-Lamberts law for 1 cm light path (b).


(3) \begin{equation*}A = \ \epsilon bc\end{equation*}

*A* denotes the absorbance value of the first excitonic peak and *c* is the unknown concentration of cadmium sulfide nanoparticles.

### Scanning Electron Microscopy

Scanning electron microscopy (SEM) was performed on a Nova NanoSEM 450 (FEI). First, 2 µl aliquots of samples were pipetted on a clean, Si wafer measuring 5 × 5 mm. The sample was allowed to dry at 80°C until the solution evaporated. Next the sample was sputter coated with 1 nm of Au/Pd with a Cressington 108 sputter coated to avoid static build-up during imaging. Finally, the sample was imaged using spot 3 and 12 kV image settings. Size analysis was completed using Fiji image analysis software.

### X-ray Diffraction (XRD) Pattern

To obtain XRD pattern, approximately 200 µl sample was spotted on a Zero diffraction plate (25 × 25 × 2 mm, Si P-type B-doped, MTI corporation) and air dried prior to subjecting it to Cu Kα radiation (15 mA, 40 V, λ = 1.5406 Å) in an X-ray diffraction system (Rigaku Ultima IV) to scan between 2θ range of 20°–70° with step size and collection time of 0.02° and 10 s respectively. Data obtained from XRD were analyzed using SmartLab Studio II software.

### Structured Illumination Microscopy

A volume of 25 ml culture of *E. coli ΔzupT* expressing p*zupT_OM_* and p*ompA_np* was used to image nanoparticle formation in cells. The sample was treated with 1 µg/ml of cadmium chloride and 0.0025 mM of sodium sulfide. The negative control did not receive cadmium or sodium sulfide. After the treatment, cells were washed thrice with 1X PBS (VWR) and fixed with 4% PFA solution (Sigma-Aldrich) for half an hour. It was followed by washing 1X PBS three times and suspension in 1 ml of 1X PBS solution. A volume of 5 µl of the suspension was spread on glass slide (3 inch X 1 inch, 1 mm) and mounted with 5 µl of mountant (20 mM Tris, pH 8, 0.5% N-propyl gallate (Sigma-Aldrich), 90% glycerol (Sigma-Aldrich)) before covering it with glass a coverslip (22 × 22 mm, 0.13–0.17 mm). Three-dimensional images of cells were acquired using Structured Illumination Microscope (DeltaVision OMX, GE Healthcare) with inverted 63X/1.42 oil immersion PlanApo N objective. A laser at 405 nm was selected for imaging nanoparticles and corresponding cell outlines were imaged via DIC. 512 × 512 sized images were recorded using sCMOS camera at exposure of 100 ms and 50% laser power. Images were taken in Z-plane with 0.125 µm per slice. Image reconstruction was later done using SoftVoRx 6.0 software.

### Mathematical Modeling and Simulations

No general analytic solution exists for describing the entire nanoparticle assembly from precursor to final nanoparticle ensemble. Classical descriptions exist, however, for individual steps. The three descriptions are that of the chemical reaction of the precursor, the nucleation of critical nuclei, and the growth of nuclei into particle ensembles. A theoretical approach to unifying these descriptions and integrating biological contexts is detailed in (Naughton & Boedicker, 2021). The model was solved numerically using the Euler forward method with MatLab. See the Supplementary Material for the code and list of parameters used (Table S3).

Briefly, the model adds biological context to simulations of nanoparticle synthesis by considering a single representative microbe that can (i) divide the simulation space into the extracellular, periplasmic, and cytoplasmic volumes; (ii) transport soluble ions among these volumes; and (iii) express substrates such as peptides that can enhance nanoparticle nucleation. Each time step, the simulation calculates the number of nuclei N of size R that form as a result of soluble chemical ions reacting to form insoluble species in the extracellular, periplasmic, and cytoplasmic space, e.g. the reaction of Cd^2+^ + S^2−^ → CdS in the periplasm. Additionally, each time step the simulation calculates the change in concentration of soluble ionic species due to transport, chemical reactions, and the adsorption into growing nanoparticles. The simulation also determines the growth of existing nanoparticles each time step, which is a function of concentration of insoluble atomic precursors like CdS. An important feature of the model is that it captures the facilitation of nucleation by proteins. The model also reflects the ability of engineered strains to modulate ion transport rates among cellular volumes, such as that for Cd^2+^ transport into the periplasm by ZupT on the outer membrane.

## Results

### Simulating CdS Nanoparticle Synthesis in *E. Coli*

The main goal of this work was to identify strategies to engineer bacterial cells for controlled production of nanomaterials from low concentrations of starting materials. To explore how biological aspects of our system influence formation of CdS nanoparticles, we deployed a previously reported simulation of biogenic nanoparticle synthesis (Naughton & Boedicker, 2021). The model captures the impact of the biological context on nanomaterial formation by simulating the redox, nucleation, and growth kinetics within a cell.

One idea was to specify the location of synthesis within a cellular compartment. To encourage nanoparticle formation, we explored how the cellular location of peptide sequences known to nucleate nanomaterials would affect nanoparticle production. Because nucleation of nanomaterials depends on the concentration of starting materials, we also simulated the influence of membrane transporters on nucleation and growth kinetics. Together these modifications, a peptide to encourage material nucleation at low concentrations of starting material and transporter proteins to increase uptake of metal ions by cells, should decrease the external concentration of metal ions required for nanoparticle synthesis.

**Fig. 1.**
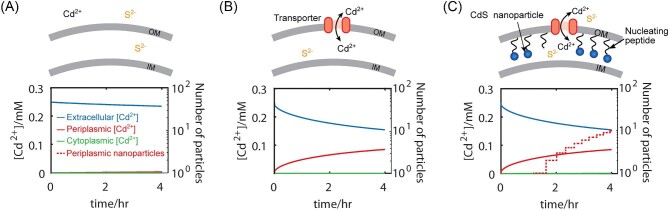
Simulation of CdS nanoparticle synthesis in the presence of a bacterial cell. As shown in the schematics, simulations were run for cells without nucleating peptides or metal ion transporters (A), with metal ion transporters only (B), and with both nucleating peptides and metal ion transporters (C). At time = 0, cadmium and sulfide ions were added to the extracellular space. The plots show the concentration of cadmium ions over time within each cellular compartment (cytoplasm, periplasm, or external) as well as the number of nanoparticles formed in the periplasm. For all conditions nanoparticles were not formed externally or in the cytoplasm.

The results of these simulations are shown in Fig. 1A–C, reporting both the concentration of Cd^2+^ and the number of synthesized nanoparticles in the cytoplasm, the periplasm, and external to the cell. We simulated the introduction of an *E. coli* cell in an environment containing external Cd^2+^ and S^2−^ ions at 0.25 mM equimolar concentration. We compared cells with and without the addition of metal ion transporters and nucleation peptide. The simulated cell can uptake precursor into periplasm and cytoplasm both through the activity of a metal ion transport protein and via diffusion through the membrane. As shown in Fig. 1A, at low concentrations of Cd^2+^, without the outer membrane metal transporter and the periplasmic peptide, no nanoparticles were produced and Cd^2+^ remains outside the cell. As shown in Fig. 1B, cell expressing the metal transporter protein in the outer membrane facilitated the uptake of Cd^2+^ ions into the periplasmic space. Despite the presence of sulfide and cadmium ions in periplasm, no particles formed as the concentration of cadmium and sulfide was too low to drive nucleation of nanoparticles. Figure 1C shows the addition of a nucleating peptide to the periplasmic space. In this case, through the combined activity of metal transporter and nucleating interfaces, the cell facilitates nanoparticles formation with periplasm (dotted red line). The model demonstrated how metal transport across the membrane and the presence of nucleating peptides could control the production nanoparticles. We next engineered a cell with these features.

### Construction and Validation of *E. Coli* Strains for CdS Synthesis

Based on our simulations, we developed two genetic modules to confer following phenotypes to *E. coli* cell: (i) expression of outer membrane transporter for cadmium ion uptake and, (ii) periplasmic expression of nucleating peptides for adsorption of cadmium sulfide molecules into nanoparticles.

The first module aims at controlled augmentation of intracellular accumulation of cadmium ions, specifically in periplasm to drive the assimilation of ions into the formation of cadmium sulfide molecules. For this, we chose to create a genetic construct expressing the *E. coli* zinc permease, *zupT*. Native ZupT protein is a zinc importer located on the inner membrane that transports divalent zinc cations from the periplasm to the cytoplasm (Grass et al., 2002). ZupT is suspected to transport many divalent cations, as many ZIP family proteins (ZRT, IRT-like Proteins) have been shown to have a broad substrate range (Guerinot, 2000). The metal-binding sites of ZupT interacted similarity with zinc and cadmium (Roberts et al., 2021), and ZupT was directly shown to enable uptake of cobalt, cadmium, and manganese by *E. coli* (Grass et al., 2005; Taudte & Grass, 2010).

ZupT thus, can potentially be employed for biosynthesis of materials with transition metals. To target synthesis within the periplasm, as shown in Fig. 1C, we modulated the cellular localization of ZupT to outer membrane through the sec-dependent pathway (Nangola et al., 2010; Thie et al., 2008). The leader peptide sequence at the N-terminal of ZupT was replaced with the leader peptide sequence of outer membrane porin, OmpA (Fig. S1A), creating *zupT_OM_*. Movement of ZupT to the outer membrane should increase flux of cadmium in the periplasm, by bringing cadmium in from the external space and reducing transport from the periplasm to the cytoplasm, due to the absence of inner membrane ZupT in the *ΔzupT* host strain.

**Fig. 2.**
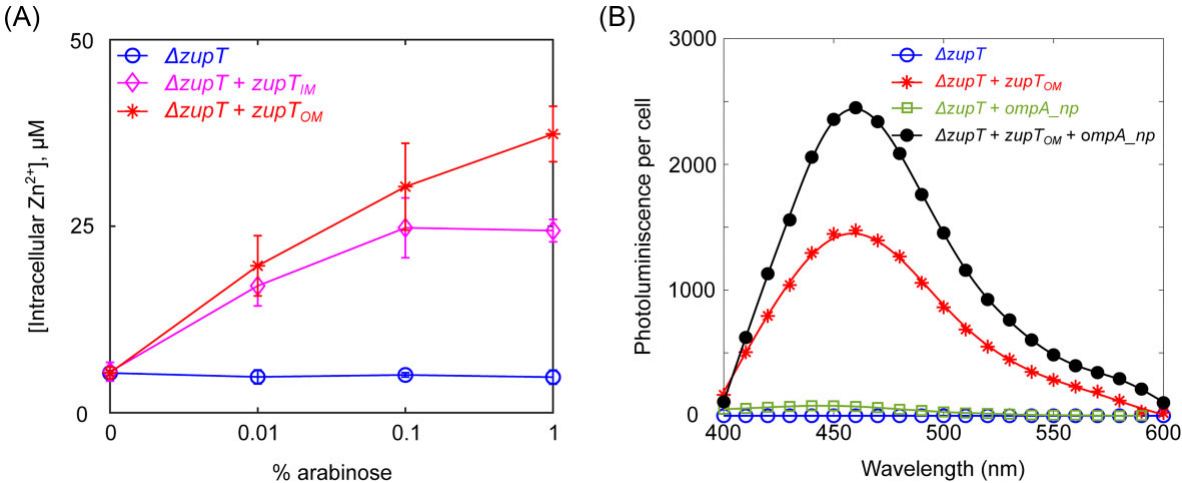
Functional analysis of outer membrane ZupT and periplasmic nucleating peptides expressed by *E. coli ΔzupT*. (A) Zinc uptake by *E. coli* cells as a function of concentration of the inducer arabinose, used to regulate expression of *zupT_IM_* and *zupT_OM_*. Intracellular zinc concentration was measured for cells without ZupT (blue solid line), cells expressing ZupT on inner membrane (*zupT_IM_*, magenta solid line), or cells expressing ZupT on outer membrane (*zupT_OM_*, red solid line). Error bars show standard deviation, each measurement was repeated in triplicate cultures. (B) Photoluminescence profile of nanomaterials harvested from strains of *E. coli* with and without the ZupT metal ion permease and the periplasmic nucleating peptide. For synthesis 0.25 mM of Na_2_S and 10 µg/ml CdCl_2_ were added to cultures.

Next, the location and function of ZupT were verified. Isolation of outer membrane fraction from the strain expressing *zupT_OM_* and subsequent SDS-PAGE analysis showed a band at around 26 kDa position, suggesting translocation of ZupT into outer membrane. While outer membrane fractions from control strains i.e. *E. coli ΔzupT* and *E. coli ΔzupT* expressing native *zupT* did not show presence of ZupT protein (Fig. S3). We then tested the strains for ZupT function by assessing their zinc uptake using a commercial kit to measure intracellular zinc. Zinc uptake is not direct evidence for uptake of other metals such as cadmium, however, the ability of cells expressing this protein to synthesize CdS nanoparticles, Fig. 2B, demonstrates that ZupT_OM_ influenced cadmium concentrations within the cell. For strains expressing *zupT_OM_* and *zupT_IM_*, zinc uptake increased with *zupT* expression (Fig. 2A). The strain expressing *zupT_OM_* accumulated more intracellular zinc than the strain expressing *zupT_IM_*. A low level of zinc uptake was observed for *ΔzupT*, likely due to other membrane proteins capable of zinc transport (Laddaga & Silver, 1985; Morozzi et al., 1986).

Once, we confirmed both activity and outer membrane localization of *zupT_OM_* construct, we validated the activity of the nucleation peptide inserted into the periplasm. The peptide sequence used here was previously identified using a yeast display system to screen for peptide sequences that bind II−VI semiconductors (Peelle et al., 2005). Here, we use the sequence ‘EEGGHHHGGEE’. The C-terminal of the OmpA protein was tagged with a hybrid peptide sequence containing linker peptide Gly_4_Ser_4_ attached to the CdS-specific peptide sequence (Fig. S1B), creating *ompA_np*. The C-terminal of OmpA faces into the periplasm. Cells expressing the nucleation peptide were used for synthesis of CdS nanoparticles.

Figure 2B shows photoluminescence spectra of material harvested from cells following nanoparticle synthesis. CdS production in cultures of *E. coli ΔzupT* with *ompA_np* and *zupT_OM_* were compared to production in the host strain *E. coli ΔzupT* and strains with only *ompA_np* or only *zupT_OM_*. The growth rates of these strains in the given growth conditions were similar (Fig. S4C). Strains were treated with 10 µg/ml of cadmium chloride and 0.25 mM of sodium sulfide. Cells excited with 365 nm light are known to show autofluorescence near 460 nm, mainly due to the presence of NADPH (Croce & Bottiroli, 2014), but increased emission at 460 nm would indicate the presence of CdS nanoparticles. The height of the peak in the photoluminescent spectra near 460 nm compared to negative controls indicates the extent of CdS nanoparticle production (Chen et al., 2021; Rai & Bokatial, 2011; Verma & Mehata, 2016). Photoluminescence increased for cells containing *zupT_OM_*, with the highest photoluminescence observed for the strain with both zupT_OM_ and *ompA_np*. Prior studies have shown extracellular biosynthesis of cadmium sulfide nanoparticle by *E. coli* cell (El-Shanshoury et al., 2012; Shivashankarappa & Sanjay, 2020), however, the extracellular medium for all four cultures did not show a detectable increase in photoluminescence compared to supernatant from cells without Cd^2+^ added (Fig. S5), which implies intracellular synthesis of CdS nanoparticles.

### Synthesis of CdS Nanoparticles at Lower Concentrations of Cadmium Chloride and Sodium Sulfide

**Fig. 3.**
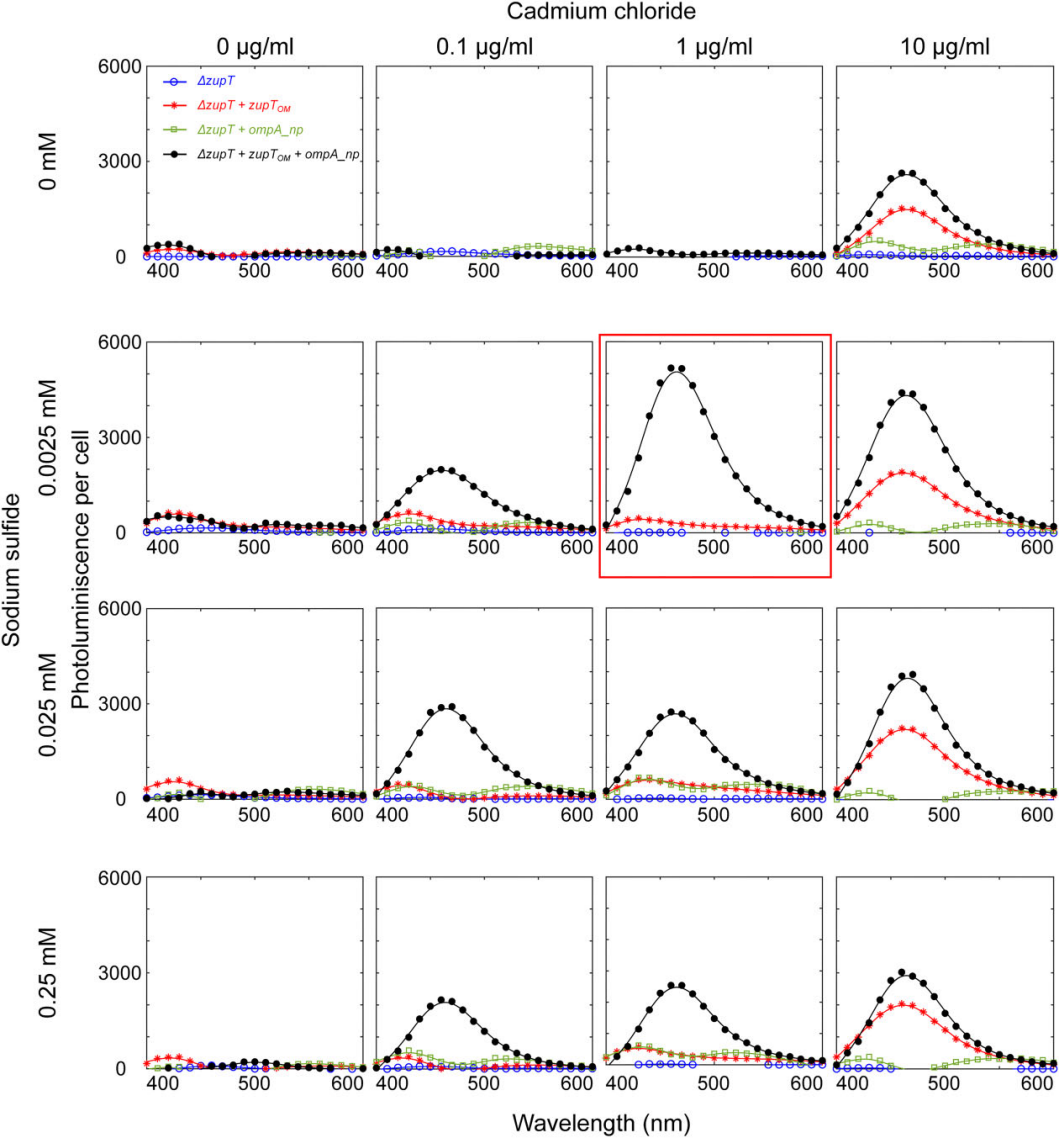
Profiling of photoluminescence of cell extract for CdS nanoparticles. Photoluminescence was measured for cell extracts isolated from *E. coli* cultures with different concentrations of added cadmium chloride and sodium sulfide and divided by respective cell density (OD_600nm_) to calculate photoluminescence per cell. Photoluminescence from cultures expressing outer membrane metal transporter ZupT and the nucleating peptide (black solid line) was compared to the host strain and cultures only expressing the peptide or the transporter (blue, red, and green solid lines). Background photoluminescence spectra of extract from the *E. coli ΔzupT* host cells without cadmium chloride and sodium sulfide was subtracted from each measurement. For each strain and condition *n* = 1.

One goal of this study was to synthesize CdS nanoparticles at concentrations of cadmium low enough not to impact cell growth. Growth measurements revealed that a reduced growth rate for all the strains in the presence of 10 ug/ml cadmium chloride and 0.25 mM sodium sulfide, (Fig. S4C and D). The growth rate was unaffected at 1 ug/ml cadmium chloride and 0.0025 mM sodium sulfide. Nanoparticles were synthesized during these growth measurements, suggesting that the presence of nanoparticles did not impact growth. Therefore, we tested the ability of our strains to synthesize CdS nanoparticles in combination of cadmium chloride concentrations between 0 and 10 µg/ml and sodium sulfide concentrations between 0 and 0.25 mM. As in Fig. 2B, we compared CdS production in *E. coli ΔzupT, E. coli ΔzupT* + *zupT_OM_, E. coli ΔzupT* + *ompA_np*, and *E. coli ΔzupT* + *zupT_OM_* + *ompA_np*. Material harvested from cell extracts were excited at 365 nm wavelength to record their respective photoluminescence at wavelengths from 400 to 600 nm. Further, to calculate photoluminescence spectra per cell, we divided photoluminescence values by cell densities measured before cell lysis. Photoluminescence values for all strains at all experimental conditions were normalized with respect to that of *E. coli ΔzupT* grown in the absence of cadmium chloride and sodium sulfide by subtraction to remove cell autofluorescence (Fig. 3, see Fig. S6 for data without background subtraction).

Among the four experimental strains, *E. coli ΔzupT* + *zupT_OM_* + *ompA_np* (solid black line) showed the most photoluminescence, with a peak near 460 nm. For cultures with 0.1 µg/ml of cadmium chloride, photoluminescence was above the background only for the strain containing *zupT_OM_* and *ompA_np*, highlighting that both the cadmium transporter and the nucleating peptide were essential for CdS synthesis at low concentrations of cadmium. The optimal photoluminescence was observed for *E. coli ΔzupT* + *zupT_OM_* + *ompA_np* treated with 1 µg/ml of cadmium chloride and 0.0025 mM of sodium sulfide (Fig. 3, red box). Note that the growth rate of all the four strains at this reaction condition is comparable to growth when no cadmium or sulfide was added (Fig. S4A and B). In the absence of cadmium chloride, no increased photoluminescence was observed for all sodium sulfide concentrations tested, whereas photoluminescence above the background was observed at 0 mM sodium sulfide and 10 µg/ml of cadmium chloride, is agreement with the absorbance measurements shown in Fig. S7. This is likely due to the low level of naturally occurring sulfide within the cell. For the range of sulfide and cadmium concentrations tested, a large increase in photoluminescence was only observed for *E. coli ΔzupT* + *zupT_OM_* and *E. coli ΔzupT* + *zupT_OM_* + *ompA_np*. This demonstrates the importance of the metal transport protein to uptake sufficient cadmium for the extracellular space to create the potential for intracellular CdS synthesis. In the absence of the nucleating peptide, and when the concentration of added cadmium was highest, nucleation may have occurred on other biomolecules or biomolecular structures.

Material harvested from cell extracts was also analyzed by optical spectroscopy from 350 to 500 nm. There was agreement between the photoluminescence and absorbance data, only samples with a photoluminescence peak above background exhibited a peak in absorbance near 375 nm (Fig. S7). The absorbance data was used to calculate particle size and extinction coefficient as explained in Yu et al., 2003 (Yu et al., 2003). The extinction coefficient was then used to estimate the concentration of cadmium sulfide nanoparticles in cellular extracts using the Beer Lambert's Law. Particle size was ∼2.8 nm and the highest concentration of CdSnanoparticles was approximately 1.6 µmole for the culture treated with 1 µg/ml of cadmium chloride and 0.0025 mM of sodium sulfide (Fig. S8, red box). These results demonstrate intracellular biosynthesis of CdSnanoparticles and highlight the critical role of the outer membrane metal transport protein and the periplasmic nucleating peptide in nanoparticle synthesis from low concentrations of reactants.

### Characterization of CdS Nanoparticles

After quantitative analysis of cellular extracts demonstrated the biogenesis of cadmium sulfide nanoparticles, we next characterized these particles. Our investigation was divided into two parts based on the objective behind the analyses. The first part deals with the confirmation of intracellular synthesis of the particles owing to the synthetic DNA parts introduced into the cell, while the second part delves into the molecular examination for size and elemental verification.

For our analysis, we used *E. coli ΔzupT* + *zupT_OM_* + *ompA_np* cultures treated with 1 µg/ml cadmium chloride and 0.0025 mM sodium sulfide (Fig. 3, red box), as we observed the highest photoluminescence at these reaction conditions.

We used fluorescence microscopy as described in previous studies, in order to observe nanoparticles (Chen et al., 2019; Stavitskaya et al., 2018; Thomas et al., 2022; Qiu et al., 2020). We fixed the whole cells with 4% PFA and subjected them to structured illumination microscopy (SIM) that has spatial resolution of 120 nm. The photoluminescence in the extracts obtained from these cells was observed upon excitation at 365 nm with an emission peak near 460 nm. Fixed cells were imaged with a 405 nm laser using 4′,6-diamidino-2-phenylindole (DAPI) emission filters. Membrane dye FM4-64 was used to aid in cell location.

**Fig. 4.**
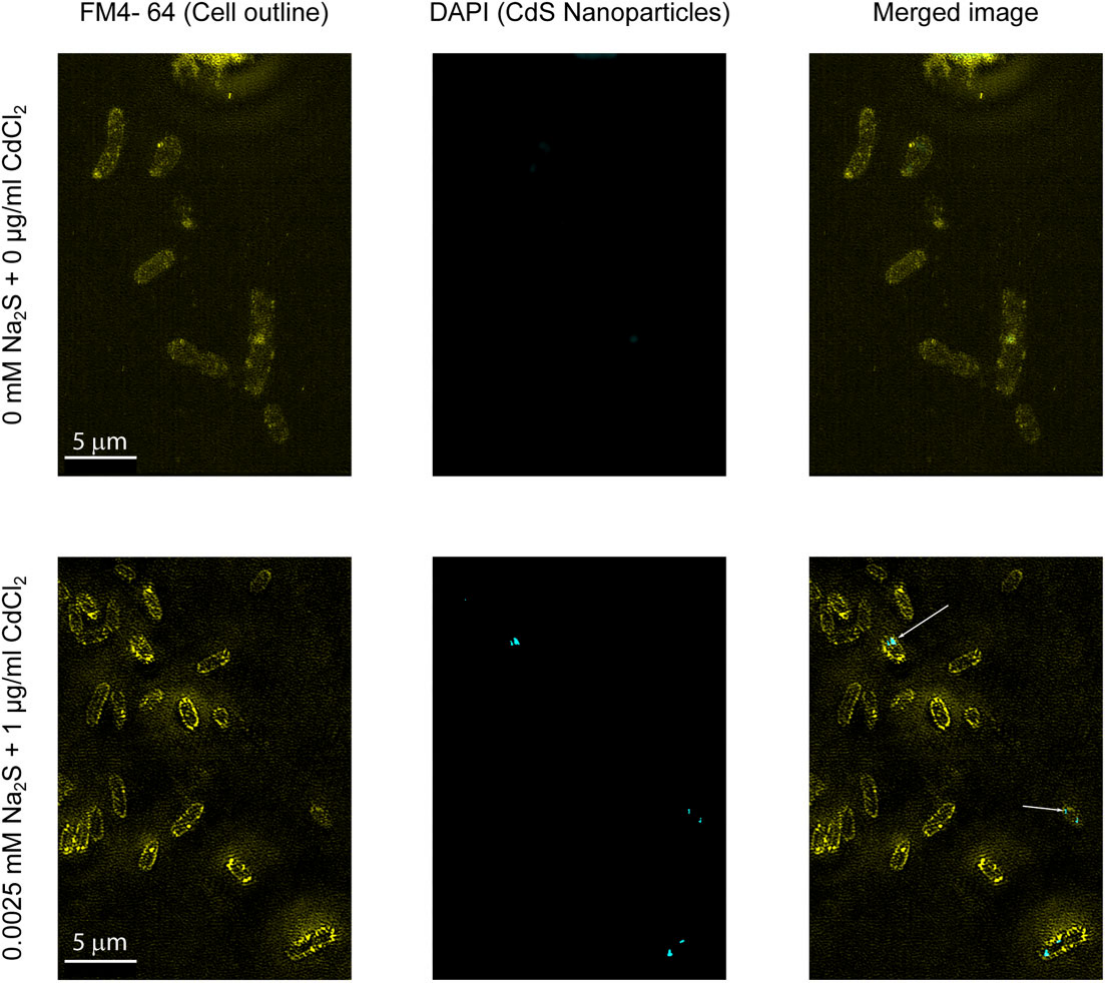
Visualization of *E. coli ΔzupT* +* zupT_OM_* +* ompA_np* cells after CdS nanoparticle synthesis using structured illumination microscopy. Samples were stained with FM4-64 to visualize cell membranes and with DAPI to visualize nanoparticles. Upper row shows cells with no sulfide or cadmium added and lower row shows cells with 0.0025 mM sulfide and 1 µ g/ml cadmium. Arrows point to nanoparticles inside of cells. 0 of 9 cells in the top images and 4 of 24 cells in the bottom images contained visible CdS nanoparticles. Scale bar: 5 µ m.

With SIM, we could observe nanoparticles within cells, as shown in Fig. 4. Objects fluorescent under DAPI illumination were observed inside of cells expressing *zupT_OM_* and the periplasmic nucleating peptide. Example images of cells with and without added cadmium and sulfide are shown in Fig. 4, and cells from 6 additional images were manually quantified. For cells with added cadmium and sulfide, 50 of 132 cells had objects that were fluorescent under DAPI illumination. These objects likely represent larger clusters of nanoparticles, and it is unclear if individual particles would be visible in the images. Cultures without added sulfide and cadmium did not contain structures that were fluorescent upon excitation at 405 nm (80 cells total observed).

**Fig. 5.**
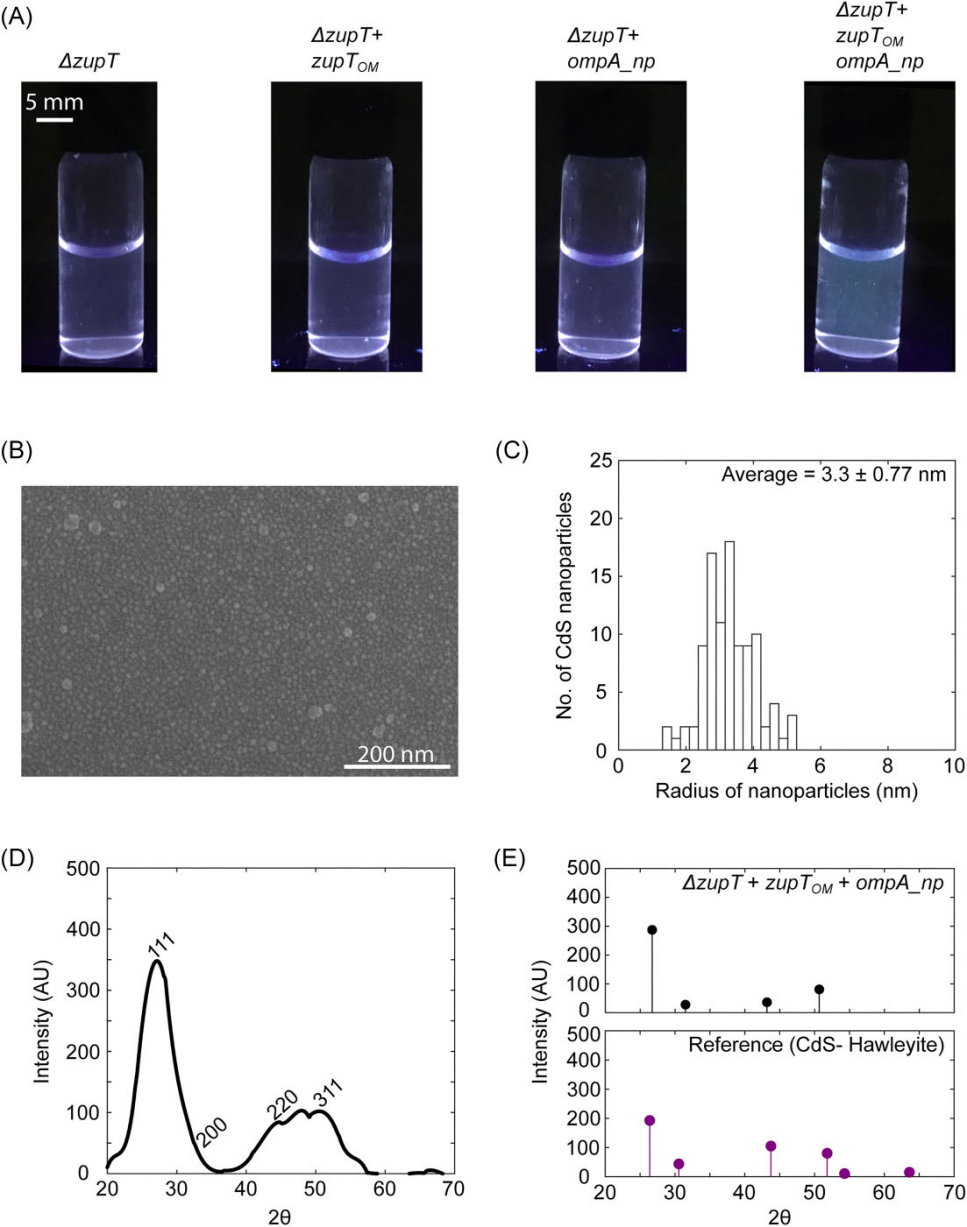
Size and elemental analysis of CdS nanoparticles. (A) Shows the cellular extract isolated from four experimental strains after illumination with UV light. (B) SEM image of CdS nanoparticles isolated from one culture of *E. coli ΔzupT* +* zupT_OM_* +* OmpA_np*. (C) Particle radii measured from the SEM image. Scale bar- 200 nm. (D) XRD analysis of the harvested nanoparticles. (E) Comparison of XRD results to reference data for CdS in Hawleyite.

Molecular analysis of harvested nanoparticles was carried out by using SEM and X-ray diffractometer. Preliminary observations obtained by illuminating cell extract to UV light, revealed that bluish glow in test sample in contrast to the control samples (Fig. 5A). The observation agreed with our photoluminescence as well as absorbance reading of the same samples (Fig. 3, Fig. S7 and Fig. S8, red box). SEM images of the extract from treated cultures of *E. coli ΔzupT + zupT_OM_ + ompA_np* showed spherically shaped nanoparticles (Fig. 5B) with size distribution ranging from 1.3 to 5.3 nm and averaging at 3.3 nm (Fig. 5C). Our SEM measurements of nanoparticle size were comparable to the size estimated from analysis of absorbance measurements. XRD analysis of the same sample exhibited prominent peaks approximately at 26.7, 31.5, 43.2, and 50.7, 2θ values (Fig. 5D). The pattern was indexed to (1 1 1), (2 0 0), (2 2 0), and (3 1 1) facets of the cubic phase of the crystal using SmartLab studio III software and was found to be comparable to the pattern exhibited by the crystal structure of Hawleyite, a sulfide mineral of cadmium. Peaks, thus observed for the experimental sample of nanoparticles, were consistent with the peaks found for reference crystals (Fig. 5E).

## Discussion

In this study, we demonstrated the ability to engineer bacterial cells to control the synthesis of inorganic nanomaterials. Methods for bacterial-assisted synthesis of metallic and inorganic nanomaterials have been known for many years. There are several naturally occurring biomolecules that facilitate nanoparticle formation (Venegas et al., 2017), including peptides and proteins that nucleate CdS and silver nanoparticles (Dameron et al., 1989; Klaus et al., 1999). The redox activity of many bacteria has also been implemented for metal reduction and subsequent nanoparticle formation (Chellamuthu et al., 2019; Dunleavy et al., 2016; Dundas et al., 2018; McFarlane et al., 2015; Zheng et al., 2020). In addition to these naturally occurring pathways, directed evolution has been used to identify peptide sequences capable of nucleating a variety of inorganic nanomaterials (Flynn et al., 2003; Krajina et al., 2018; Sweeney et al., 2004; Thai et al., 2004). Our approach also utilized a metal transport protein, ZupT, from *E. coli*. Although first identified in the context of zinc transport (Grass et al., 2002), prior work demonstrated the ability of this membrane permease to transport many divalent metals, including copper, manganese, and cadmium (Grass et al., 2005). This study shows how the engineering of metal transport proteins can be used to control nanoparticle synthesis in bacteria. Recent work reported that deletion of a metal export protein, ZntA, increased Cd concentrations inside of cells increasing CdS nanoparticle formation (Zhu et al., 2021). As predicted by the simulation in Fig. 1 and demonstrated in Fig. 3, the combination of this metal transport protein and nanoparticle nucleating peptides was critical to nanoparticle synthesis at low concentrations of starting materials. These results demonstrate how modifying multiple aspects of nanoparticle nucleation and growth within cells can increase the precision and control of biogenic nanoparticle synthesis.

Here, nanoparticle synthesis within cells was controlled via a combination of metal ion transport and material nucleation, but biology offers a variety of strategies to interface with inorganic material synthesis. In general, the ability to synthesize nanomaterials depends on the redox state and concentration of reactants as well as how these concentrations change with time. Material synthesis in cells, which introduces membrane separated compartments, gives new possibilities to manipulate these concentrations spatially. Prior work has shown that the location of redox activity could impact the location of palladium particle synthesis (Dundas et al., 2018). This work also shows how the location of biomolecules for heterogeneous nucleation of materials and transport proteins that facilitate metal ion exchange between such compartments can be used to regulate nanomaterial synthesis in cells. ZupT is only one pathway that contributes to metal uptake by bacteria. ABC transporters, TonB dependent transport, and other metal permeases are involved in the transports of metals across membranes. Metal chaperone proteins also help deliver metals to specific locations within cells and could influence nanomaterial synthesis (Ma et al., 2009). These strategies should be implemented to give more precise control over metal concentrations and the nucleation and growth of nanomaterials within cells. The yield of nanoparticles is relevant to real-world applications. Figure S8 reports estimated nanoparticle yields for this study. Based on these calculated yields, the reactions converted approximately 60–90% of added cadmium into CdS nanoparticles, similar to the 85% cadmium yield reported for biogenic synthesis via *Rhodobacter sphaeroides* (Bai et al., 2009). The reactions reported up to 0.2 mg of nanoparticles in 25 ml of cell culture, which is a 55 µ M concentration of nanoparticles. These yields are lower than reported for both chemical and biological synthesis of CdO nanoparticles, which were 1756.8 and 805.83 µ M concentrations, respectively (Nasrullah et al., 2020). Further optimization of the strains and reaction conditions would be needed to increase nanoparticle yields for biogenic synthesis of CdS using the strains reported here.

By colocalizing the biomolecules involved in the nucleation and growth process, we demonstrated the synthesis of CdS nanoparticles from low concentrations of reactants. In fact, as shown in Supplementary Fig. 3, the cell growth rate was not reduced at the concentrations of cadmium and sulfide used during nanoparticle synthesis. The potential for living cells to interact with and direct the synthesis of nanoparticles could enable future advances and new applications for nanomaterials synthesis within cells. Living cells are able to monitor and respond to changes inside the cell or in the external environment, potentially enabling feedback during synthesis. Biology has already evolved metal responsive transcription factors (Liu et al., 2022), which could repress or activate genes involved in material synthesis in response to the concentration of metal ions. Reaction conditions that are not toxic to the cell are required for cells to sense and respond to their environment during synthesis. Synthesis at lower reactant concentrations, as demonstrated here using nucleating peptides and metal ion transporters, will be critical for synthetic schemes that require live cells.

## Conclusion

In summary, we engineered strains of bacteria for synthesis of inorganic nanomaterials. A permease for metal ion transport through the membrane and a peptide sequence evolved to nucleate nanoparticle crystals were expressed in an *E. coli* host strain. This strain was capable of synthesizing intracellular cadmium sulfide nanoparticles from low concentrations of starting materials, as predicted by a computational model of nanoparticle nucleation and growth within cell cultures. Expression of the metal ion permease and the periplasmic nucleating peptide were critical to nanoparticle synthesis at low concentrations of cadmium and sulfide, and particles were only detected within cells. This approach demonstrates the ability to engineer bacterial cells for precise biogenesis of inorganic nanomaterials.

## Supplementary Material

kuad020_Supplemental_Files

## Data Availability

The raw data supporting the conclusion of this article will be made available upon request. All the requests should be directed to the lead contact, Dr. James Q. Boedicker (boedicke@usc.edu).
