# Supplementary material for: Utilizing a divalent metal ion transporter to control biogenic nanoparticle synthesis
Source: J Ind Microbiol Biotechnol. 2023 Aug 16;50(1):kuad020. doi: 10.1093/jimb/kuad020 (PMC10481092; doi:10.1093/jimb/kuad020)
Supplement: kuad020_Supplemental_Files [file kuad020_supplemental_files.zip › Gangan_JIMB_2023_SI.docx]

Supplementary Material

Utilizing a divalent metal ion transporter to control biogenic nanoparticle synthesis

MS Gangan, KL Naughton, and JQ Boedicker

# Supplementary Figures and Tables

| Name of the plasmid | Reference |
| --- | --- |
| p*zupT_IM_* | This study |
| p*zupT_OM_* | This study |
| p*ompA_np* | This study |

Table 1: Plasmids used in this study.

| Name of the primer | Sequence (5’-3’) |
| --- | --- |
| Construction of p*zupT_IM_* | |
| pBAD24_F | AATTGGTTAAGGCTGTTTTGGCGGATGAG |
| pBAD24_R | GTACTGACATGGTGAATTCCTCCTGCTAG |
| zupT_F | GGAATTCACCATGTCAGTACCTCTCATTCTGAC |
| zupT_R | CAAAACAGCCTTAACCAATTCCCGCCGTTTG |
| Construction of p*zupT_OM_* | |
| pzupT_IM__F | ACTGGCTGGTTTCGCTACCGTAGCGCAGGCCATGTCAGTACCTCTCATTC |
| pzupT_IM__R | GCCACTGCAATCGCGATAGCTGTCTTTTTCATTGAATTCCTCCTGCTAGC |
| Construction of p*ompA_np* | |
| pDSG372_F | GCAGGCTTAATAATACTAGTAGCGGCCG |
| pDSG372_R | TCTTTTTCATCTAGTATTTCTCCTCTTTCTC |
| ompA_F | GAAATACTAGATGAAAAAGACAGCTATCGCG |
| ompA_R | ACTAGTATTATTAAGCCTGCGGCTGAGTTAC |
| Insertion of nucleating sequence | |
| pompA_np1_F | TAAACACAAACACTGGCACTGGTAATACTAGTAGCGGCCGC |
| pompA_np1_R | GAAGAAGAAGAACCACCACCACCAGCCTGCGGCTGAGTTAC |
| pompA_np2_F | TCGCCGCCACCACCACCGCCGCTAATACTAGTAGCGGCCGC |
| pompA_np2_R | GAAGAAGAAGAACCACCACCACCAGCCTGCGGCTGAGTTAC |
| pompA_np3_F | TGGTCACGGTCACGGTCACGGTTAATACTAGTAGCGGCCGC |
| pompA_np3_R | GAAGAAGAAGAACCACCACCACCAGCCTGCGGCTGAGTTAC |
| pompA_np4_F | AGGTGGTCACCACCACGGTGGTGAAGAATAATACTAGTAGCGGCCGC |
| pompA_np4_R | TCTTCAGAAGAAGAAGAACCACCACCACCAGCCTGCGGCTGAGTTAC |
| Change of antibiotic from Kan^r^ to Chlor^r^ | |
| pompA_np_kan_F | CGGGGCGTAATCAGAATTGGTTAATTGGTTG |
| pompA_np_kan_R | TTTTCTCCATAACACCCCTTGTATTACTG |
| Chlor_F | AAGGGGTGTTATGGAGAAAAAAATCACTGGATATACCACCGTTGATATATCCC |
| Chlor_R | CCAATTCTGATTACGCCCCGCCCTGCCA |

Table 2: Primers used in this study.

## Supplementary Figures


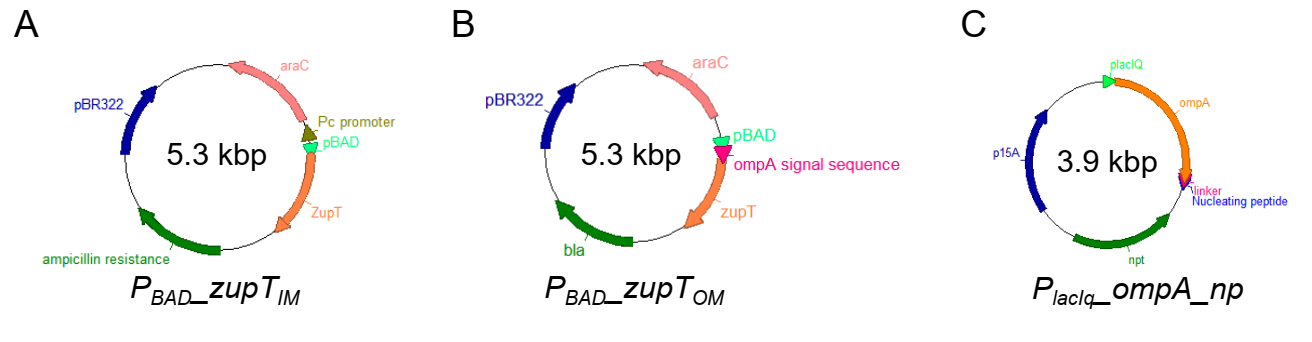
**Supplementary Figure 1.** Plasmid maps: Plasmid constructs of (A) native, (B) outer membrane variant of *zupT* expressed under arabinose control and (C) *ompA* ORF tagged with nucleating peptide and cloned under PlacI_q_.


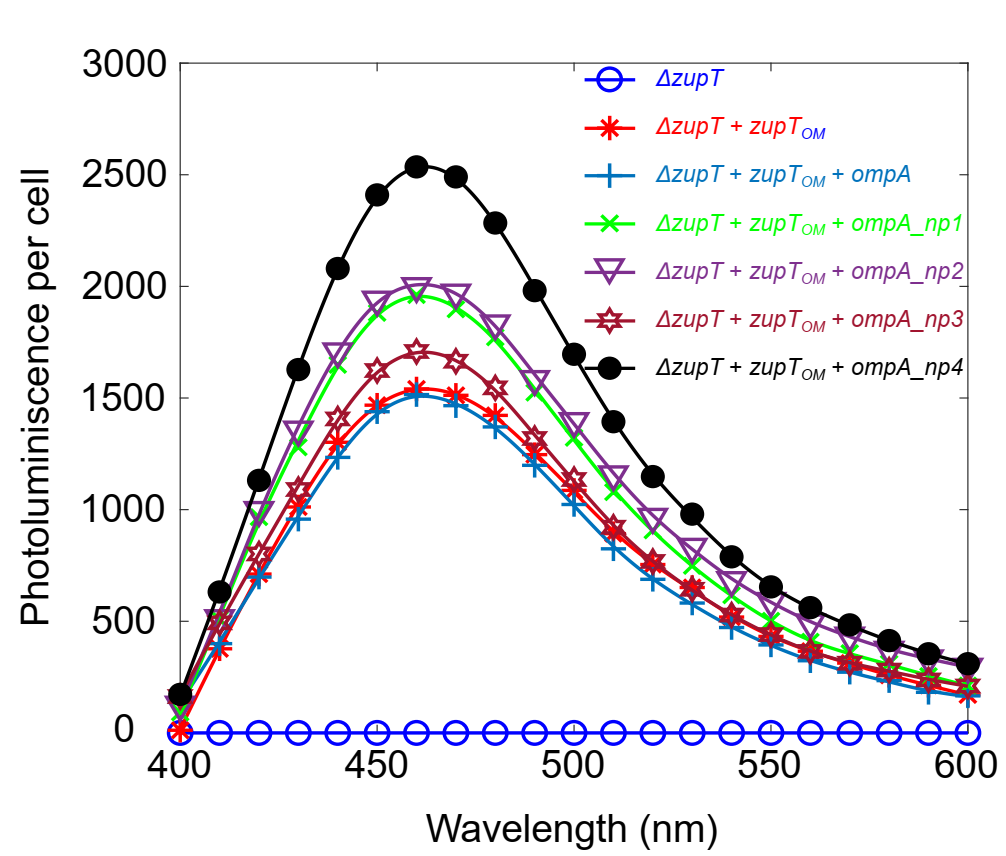


**Supplementary Figure 2.** Screening of nucleating peptides for the preparation of CdS nanoparticles. Individual cultures of *E. coli* *ΔzupT* + *zupT_OM_* were cloned to express different nucleating peptides with their respective amino acid sequences as follows: np1- KHKHWHW; np2- RRHHHRR; np3- GHGHGHG; np4- EEGGHHHGGEE. After their incubation with 0.25 mM of sodium sulfide followed by the addition of 10 µg/ml CdCl_2_,they were tested for intracellular photoluminescence as shown in the figure. Note that the highest photoluminescence was observed for *ΔzupT* + *ompA_np4* and hence was selected as a test strain for the studies discussed here. The strain has been referred to as *ΔzupT* + *ompA_np* throughout the main text*.*


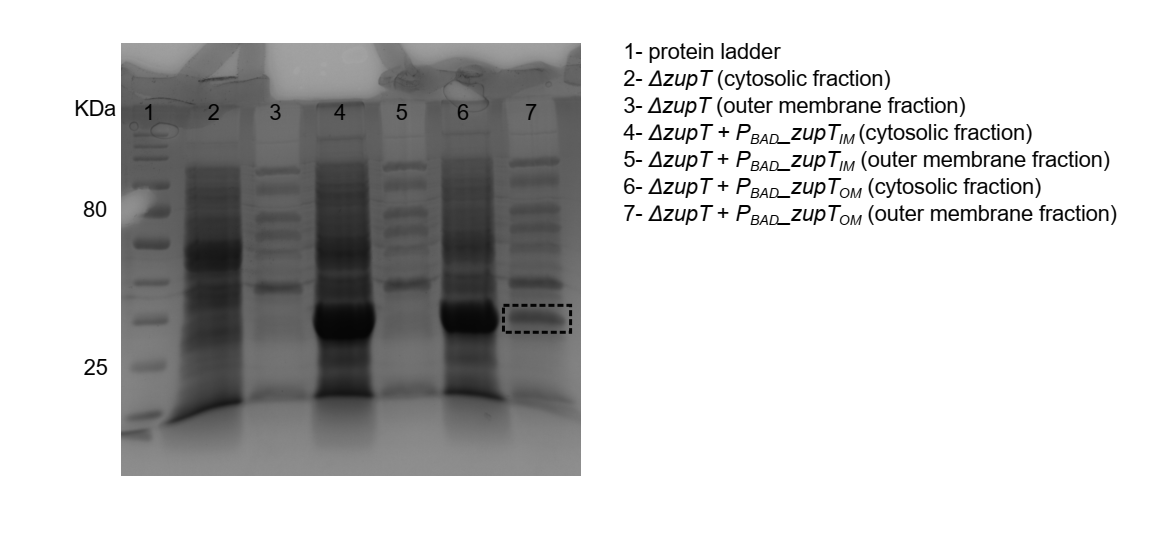


**Supplementary Figure 3.** Confirmation of outer membrane localization of ZupT. Protein gel of *E. coli ΔzupT* + *zupT_OM_* outer membrane extract shows protein band at approximately 26 KDa (dotted black box), confirming the expression and insertion of synthetic ZupT protein into the outer membrane.


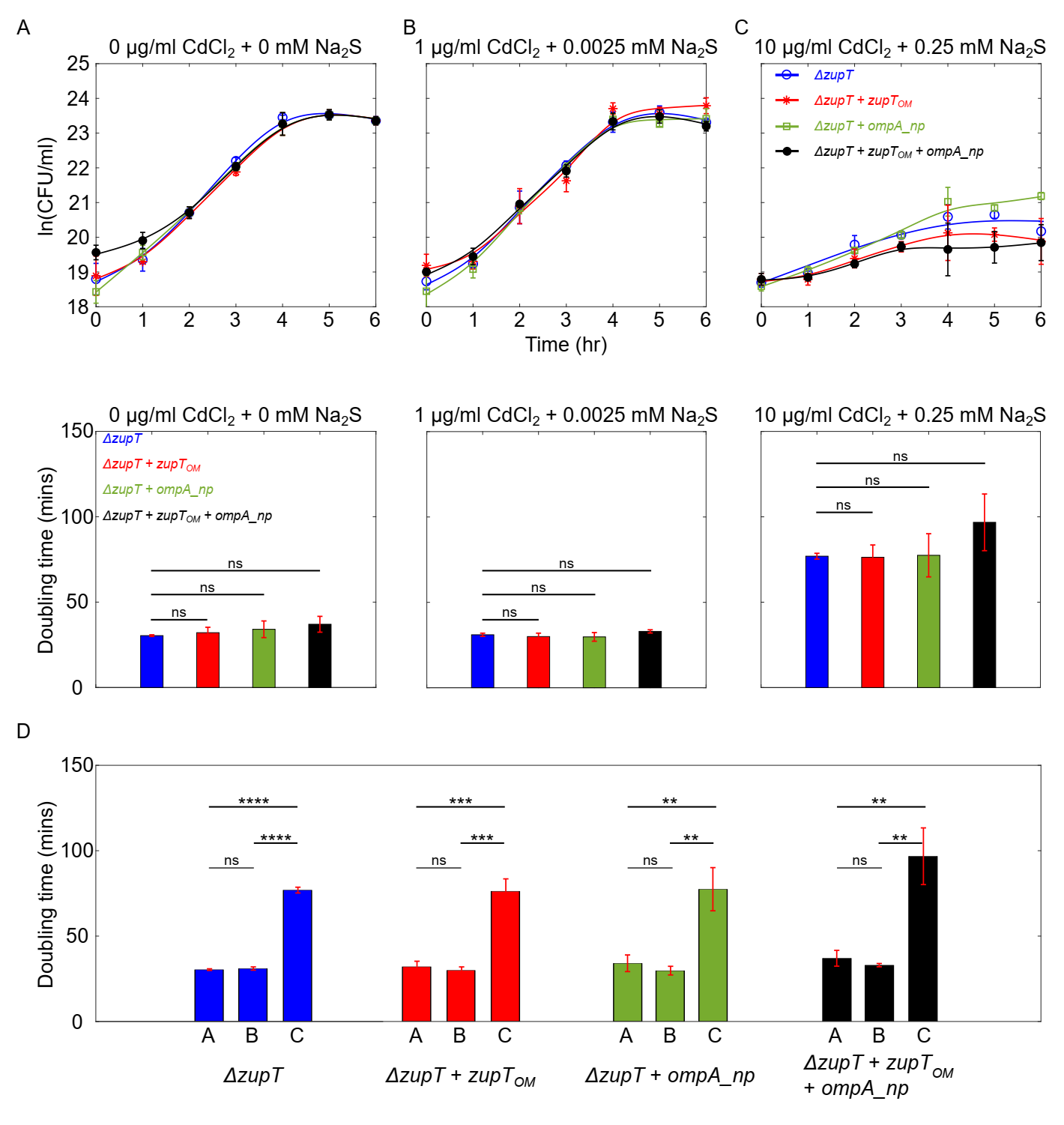


**Supplementary Figure 4.** Growth analysis of strains used in experiments. Experimental strains were grown in LB containing (A) 0 µg/ml cadmium chloride and 0 mM sodium sulfide, (B) 1 µg/ml cadmium chloride and 0.0025 mM sodium sulfide, and (C) 10 µg/ml cadmium chloride and 0.25 mM sodium sulfide. Upper panels report the cell density over time. Lower panels show the calculated doubling time of the cultures. Doubling time of each strain was compared with that of *E. coli* *ΔzupT* (host strain) for respective growth conditions using Student’s t- test. The p- value for each pair considered in this figure was found to be greater than 0.05. Hence, the difference between their doubling time/ growth rate was deemed to be non- significant (ns). (D) Doubling time of each strain across the reaction conditions have been compared with student’s t- test. ns: p > 0.05; **: p ≤ 0.01; ***: p ≤ 0.001; ****: p ≤ 0.0001. For each condition n=3.


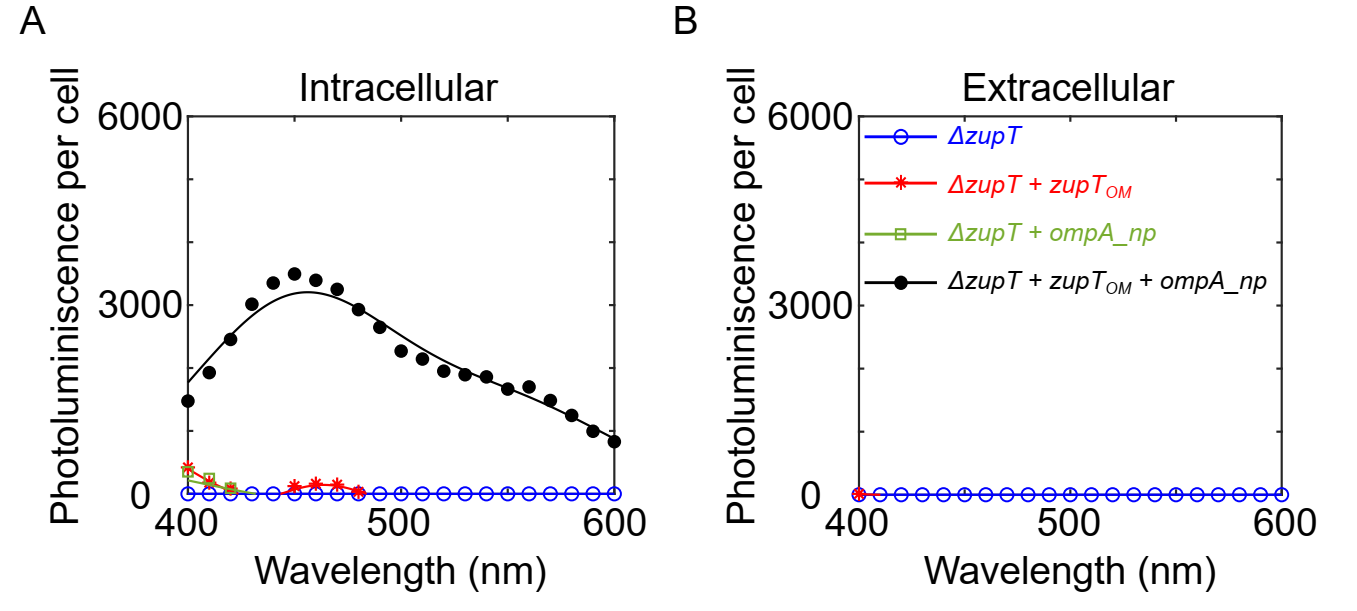


**Supplementary Figure 5.** Quantification of cadmium sulfide nanoparticles in the extracellular and intracellular environments. Photoluminescence was measured after excitation at 365 nm. Intracellular samples were cellular extract and extracellular samples were culture supernatant.


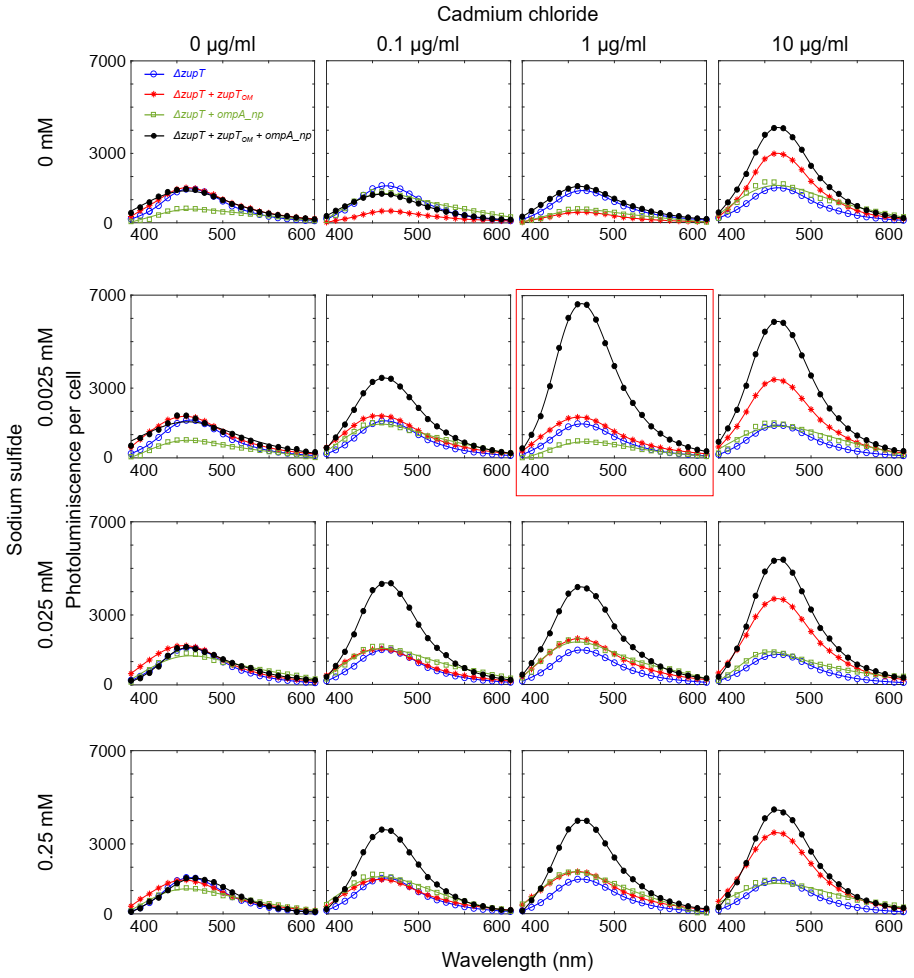


**Supplementary Figure 6.** Photoluminescence measurements of cell extract to detect cadmium sulfide nanoparticles. Raw values of photoluminescence of cell extract isolated from *E. coli* cultures were measured for different concentrations of cadmium chloride and sodium sulfide. Extracts from cultures of *E. coli* expressing outer membrane ZupT and the nucleating peptide np4 (black line) where compared to strains expressing only the outer membrane ZupT (red line), only the peptide (green line), or the host strain (blue line). For each strain and condition n=1.


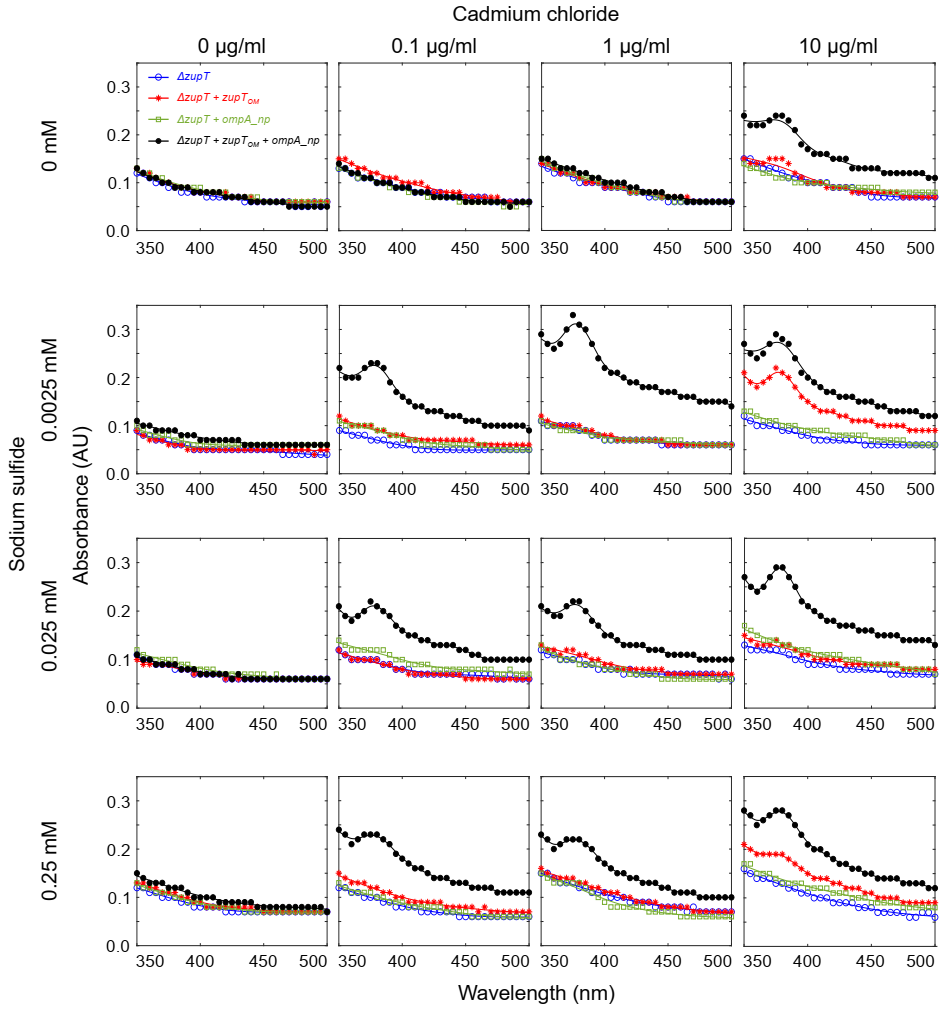


**Supplementary Figure 7.** Absorbance of cell extract at wavelengths ranging from 350 nm to 500 nm. Extracts from cultures of *E. coli* expressing outer membrane ZupT and the nucleating peptide (blackline) were compared to strains expressing only the outer membrane ZupT (red line), only the peptide (green line), or the host strain (blue line). For each strain and condition n=1.


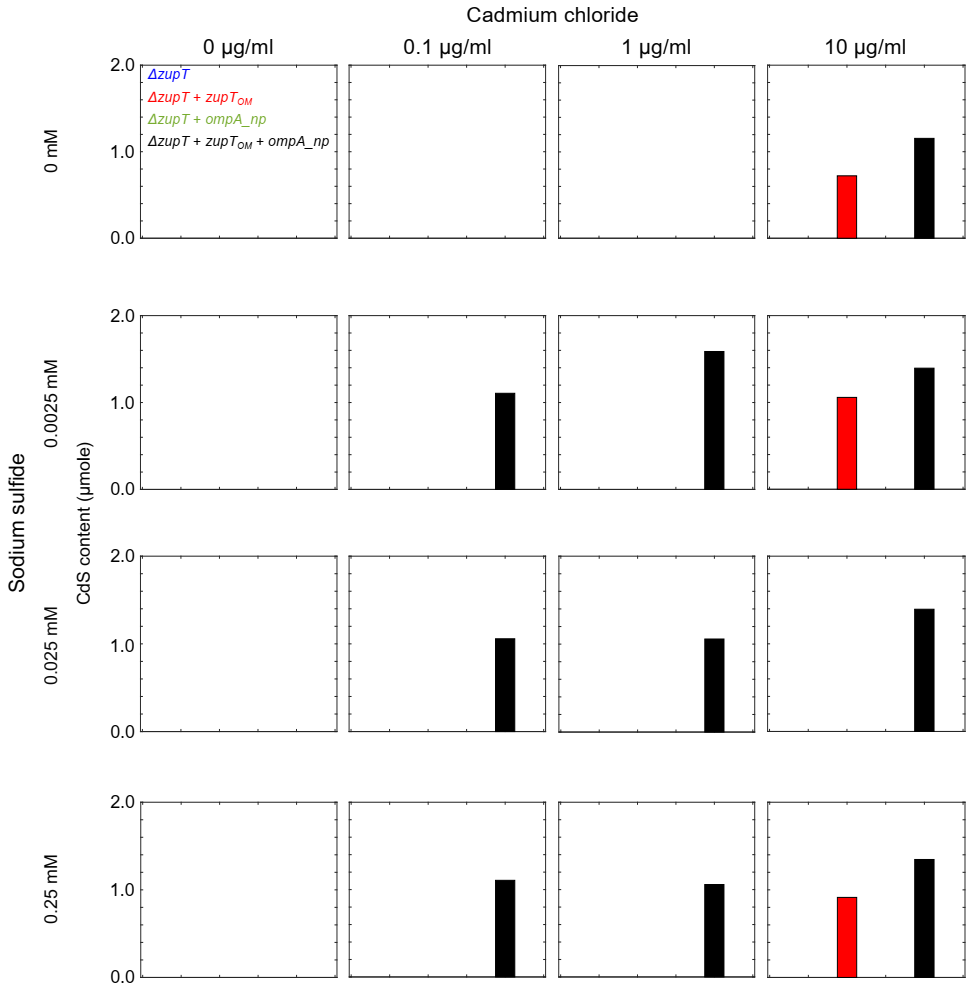


**Supplementary Figure 8.** Quantification of cadmium sulfide nanoparticles in cellular extracts. Concentration of cadmium sulfide nanoparticles was calculated for the samples exhibiting first exciton in absorbance reading using the formula explained in material and methods. Samples without measurable absorbance over the background indicated a nanoparticle yield of 0. Yield is from 25 mL cultures of cells.
