# Supplementary material for: Utilizing a divalent metal ion transporter to control biogenic nanoparticle synthesis
Source: J Ind Microbiol Biotechnol. 2023 Aug 16;50(1):kuad020. doi: 10.1093/jimb/kuad020 (PMC10481092; doi:10.1093/jimb/kuad020)
Supplement: kuad020_Supplemental_Files [file kuad020_supplemental_files.zip › Gangan_supplementary_code.docx]

close all % closes all figures

clear all % clears all values

% variables to change, number of metal ion transporters in the

% outermembrane and the number of nucleating peptides in the periplasm

OMtrans = 400; % or 0

peri_nuc = 400; % or 0

% --------- Simulation context-----------

transport.true = 1; % make = 1 for protein metal ion transporters

transport.leaky = 1; % make = 1 to allow diffusion through membrane

nucleation.true = 1; % make = 1 for the presence of nucleating peptides

% --------- DEFINE TIME STEPS ---------

h = 10; % time step in seconds

Nsteps = 4; % number of hours of simulated time

Nsteps = Nsteps*3600/h; % convert to seconds

time = (1:Nsteps)*h/3600; % time in hours for plot

% --------- INITIALIZE EMPTY ARRAYS ---------

Particles_homo(1).ext_radius = [];

Particles_het(1).ext_radius = [];

Particles_homo(1).ext_num =0;

Particles_het(1).ext_num =0;

Particles_homo(1).peri_radius = [];

Particles_het(1).peri_radius = [];

Particles_homo(1).peri_num =0;

Particles_het(1).peri_num =0;

Particles_homo(1).cyto_radius = [];

Particles_het(1).cyto_radius = [];

Particles_homo(1).cyto_num =0;

Particles_het(1).cyto_num =0;

dN_homo = zeros(round(Nsteps),3); % change in number of particles (homogeneous nucleation)

dN_het = zeros(round(Nsteps),3); % change in number of particles (heterogeneous nucleation)

rcrit_homo = zeros(round(Nsteps),3); % critical radius (homogeneous nucleation)

rcrit_het = zeros(round(Nsteps),3); % critical radius (heterogeneous nucleation)

y = zeros(round(Nsteps),3,3); % molar concentration of precursor, monomer, monomer to nuclei, monomer to growth

dydx = zeros(round(Nsteps),3,3); % rate of consumption of above things

S = zeros(round(Nsteps),3); % oversaturation at each time step

% Colormap for plotting

c = [0.01, 0.34, 0.56; % Ext, homo

0.44, 0.71, 0.89; % Ext, hetero

0/255, 102/255, 0/255; % peri, homo

119/255, 172/255, 48/255; % peri, hetero

0.64, 0.08, 0.18; % cyto, homo

0.93, 0.68, 0.58];% cyto, hetero

vol = [100e-15; 1/3*1e-15; 2/3*1e-15]; % in L units, volume of cells ~1fL and 1/3 of that is periplasmic BNID 104052, PMID 824274 p.334

% Chemical rxn rate

k1 = 20*10^-3; % 1/(M s)

% Volume of atomic monomer

v0 = 4.9769*10^-29; % in m^3

% Radius of atomic monomer

r0 = (3*v0/(4*pi))^(1/3); % radius of CdS "atom" is m

% Temperature of reaction (input in C, converted to K, then kT calculated)

T = 310.15; % temperature in Kelvin 298 is room temp, 310.15 is 37

kT = 1.38*10^-23 * T; % Boltzman's constant * temperature in Kelvin

% Diffussion coefficient of atomic monomer, related to particle growth

D = 10^-9; % esitmated diffussion coefficient m^2/s of typical O2,C02,KCl in H20, BioNumber 104440,102625 http://book.bionumbers.org/what-are-the-time-scales-for-diffusion-in-cells/

% Avogadro's Number

Na = 6.022*10^23; % Avogadros number, atoms/mole

% Viscosity of medium and its temperatue dependence

viscosity = (2.414e-5)*10^(247.8/(T-140)); % viscocity versus temperature

% parameter from Talapin et al 2002 that appears in dR/dt growth equation

alpha = 0.5; % for growth rate from Talapin et al 2002

% Basal growth rate of material.

kgr = 2e-5; % growth rate m/s used in Chen et al 2015

% Interfacial energy of C0

gamma = 0.125; % used in Talapin et al 2002

% Solubility of C0

C0_sat = 1*10^-7; % Saturation conc./ solubility

% --------------- PRECURSOR PARAMETERS in milli-moles/L ---------------

% (time point, chemical type, compartment)

y(1,1,:) = [.25; 0.0; 0.0]*1e-3; % moles/L of precursor Cd2+,

y(1,2,:) = [.25; 0.0; 0.0]*1e-3; % moles/L of precursor S2-

y(1,3,:) = [0.0; 0.0; 0.0]*1e-3; % moles/L of monomer, CdS

% --------------- TRANSPORT PARAMETERS ---------------

if transport.true == 1

% Column 1 is outer membrane, column 2 is inner memrane

N_trans = [OMtrans 0; % for Species 1

0 0; % for Species 2

0 0]; % for Species 3

else

N_trans = zeros(3,2);

end

% Assume Michaelis-Menton Kinetics, which uses Vmax (max rate) and Km (conc. of half-maximum rate of substrate/ precursor)

Vmax = [1.995e-9; % ion transport rate Cd2+

0; % ion transport S2-

0]; % ion transport the insoluble metal species

Km = [1e-6; % mole/L

1e-6; % S2-

1e-6]; % insoluble monomers

% LEAKY TRANSPORT PARAMETERS ---------------

% Note: In general, transport can be leaky despite no transport proteins being expressed.

if transport.leaky == 1

leaky = [0.000001 0.000001; % leaky transport for Species 1

.0001 .0001; % leaky transport for Species 2

0 0]; % leaky transport for Species 3

else

leaky = zeros(3,2);

end

% --------------- NUCLEATION PARAMETERS ---------------

if nucleation.true == 1

nuc_sites = [0; peri_nuc; 0];% number of nucleating sites

theta = 0.05; % amount by which gamma reduced = gamma/gamma_0

else

nuc_sites = [0; 0; 0];

theta = 1.0;

end

initial_nuc_sites = nuc_sites;

nucleation.enforce_number = 1; % this turns ON (1) or OFF (0) a finite number of sites

% note that if = 1, the sites fill up then heteronucleation ends

x71 = ['Extracellular Nuc Sites = ' num2str(nuc_sites(1))];

x72 = ['Periplasmic Nuc Sites = ' num2str(nuc_sites(2))];

x73 = ['Cytoplasmic Nuc Sites = ' num2str(nuc_sites(3))];

for i=1:Nsteps-1

% --------------- START CALCULATING THINGS ---------------

% Update moles

y(i+1,1,:) = y(i,1,:) + h*dydx(i,1,:); % Cd2+

y(i+1,2,:) = y(i,2,:) + h*dydx(i,2,:); % e.g. S2-

y(i+1,3,:) = y(i,3,:) + h*dydx(i,3,:); % e.g. CdS

% Transport and monomer formation

% Precursor 1, e.g. Cd2+

dydx(i+1,1,:) =[-k1*y(i,1,1)*y(i,2,1) - (N_trans(1,1)*Vmax(1)*y(i,1,1))/(Km(1)+y(i,1,1)) + (N_trans(1,1)*Vmax(1)*y(i,1,2))/(Km(1)+y(i,1,2))-leaky(1,1)*y(i,1,1)+leaky(1,1)*y(i,1,2);

-k1*y(i,1,2)*y(i,2,2) - (N_trans(1,1)*Vmax(1)*y(i,1,2))/(Km(1)+y(i,1,2)) + (N_trans(1,1)*Vmax(1)*y(i,1,1))/(Km(1)+y(i,1,1))-leaky(1,1)*y(i,1,2)+leaky(1,1)*y(i,1,1)...

- (N_trans(1,2)*Vmax(1)*y(i,1,2))/(Km(1)+y(i,1,2)) + (N_trans(1,2)*Vmax(1)*y(i,1,3))/(Km(1)+y(i,1,1))-leaky(1,2)*y(i,1,2)+leaky(1,2)*y(i,1,3);

-k1*y(i,1,3)*y(i,2,3) - (N_trans(1,2)*Vmax(1)*y(i,1,3))/(Km(1)+y(i,1,3)) + (N_trans(1,2)*Vmax(1)*y(i,1,2))/(Km(1)+y(i,1,2))-leaky(1,2)*y(i,1,3)+leaky(1,2)*y(i,1,2)];

% Precursor 2, e.g. S2-

dydx(i+1,2,:) =[-k1*y(i,1,1)*y(i,2,1) - (N_trans(2,1)*Vmax(2)*y(i,2,1))/(Km(2)+y(i,2,1)) + (N_trans(2,1)*Vmax(2)*y(i,2,2))/(Km(2)+y(i,2,2))-leaky(2,1)*y(i,2,1)+leaky(2,1)*y(i,2,2);

-k1*y(i,1,2)*y(i,2,2) - (N_trans(2,1)*Vmax(2)*y(i,2,2))/(Km(2)+y(i,2,2)) + (N_trans(2,1)*Vmax(2)*y(i,2,1))/(Km(2)+y(i,2,1))-leaky(2,1)*y(i,2,2)+leaky(2,1)*y(i,2,1)...

- (N_trans(2,2)*Vmax(2)*y(i,2,2))/(Km(2)+y(i,2,2)) + (N_trans(2,2)*Vmax(2)*y(i,2,3))/(Km(2)+y(i,2,1))-leaky(2,2)*y(i,2,2)+leaky(2,2)*y(i,2,3);

-k1*y(i,1,3)*y(i,2,3) - (N_trans(2,2)*Vmax(2)*y(i,2,3))/(Km(2)+y(i,2,3)) + (N_trans(2,2)*Vmax(2)*y(i,2,2))/(Km(2)+y(i,2,2))-leaky(2,2)*y(i,2,3)+leaky(2,2)*y(i,2,2)];

% Monomer 1, e.g. CdS0

dydx(i+1,3,:) =[+k1*y(i,1,1)*y(i,2,1)- (N_trans(3,1)*Vmax(3)*y(i,3,1))/(Km(3)+y(i,3,1)) + (N_trans(3,1)*Vmax(3)*y(i,3,2))/(Km(3)+y(i,3,2))-leaky(3,1)*y(i,3,1)+leaky(3,1)*y(i,3,2);

+k1*y(i,1,2)*y(i,2,2) - (N_trans(3,1)*Vmax(3)*y(i,3,2))/(Km(3)+y(i,3,2)) + (N_trans(3,1)*Vmax(3)*y(i,3,1))/(Km(3)+y(i,3,1))-leaky(3,1)*y(i,3,2)+leaky(3,1)*y(i,3,1)...

- (N_trans(3,2)*Vmax(3)*y(i,3,2))/(Km(3)+y(i,3,2)) + (N_trans(3,2)*Vmax(3)*y(i,3,3))/(Km(3)+y(i,3,1))-leaky(3,2)*y(i,3,2)+leaky(3,2)*y(i,3,3);

+k1*y(i,1,3)*y(i,2,3) - (N_trans(3,2)*Vmax(3)*y(i,3,3))/(Km(3)+y(i,3,3)) + (N_trans(3,2)*Vmax(3)*y(i,3,2))/(Km(3)+y(i,3,2))-leaky(3,2)*y(i,3,3)+leaky(3,2)*y(i,3,2)];

% Supersaturation

S(i,:) = y(i,3,:)/C0_sat;

% ==================== Nucleation ====================

%

for ll = 1:3 % loop through regions

if S(i,ll) > 1 % check if anything can happen

% homogenous nucleation always occurs if S > 1

Rcap = 2*gamma*v0/kT; % Capillary radius

rcrit_homo(i,ll) = Rcap/log(S(i,ll)); % radius of new nuclei

% Prevent non-physical nuclei forming

if rcrit_homo(i,ll) <= r0

rcrit_homo(i,ll) = r0; % if rcrit < radius of single atoms, force nuclei to be a single atom

end

vcrit_homo = (4*pi/3)*rcrit_homo(i,ll)^3; % volume of new nuclei

dG = 16*pi*gamma^3*v0^2/(3*(kT)^2*log(S(i,ll))^2);

% NUCLEATION RATE (HOMOGENOUS)

knuc_homo = (y(i,3,ll)*Na*vol(ll))*(kT/(3*pi*(2*r0)^3*viscosity))*exp(-dG/kT);

dN_homo(i,ll) = floor(h*(knuc_homo));

% determine if there is enough CdS to nucleate all the particles

dN_homo(i,ll) = min([dN_homo(i,ll), floor((y(i+1,3,ll)*vol(ll)*Na)/(vcrit_homo/v0))]);

% === update numbers of homogeneously nucleated NP

if ll == 1

Particles_homo(i+1).ext_radius = [[Particles_homo(i).ext_radius], rcrit_homo(i,ll)*ones(dN_homo(i,ll),1)']; % add new nuclei of radius rcrit

Particles_homo(i+1).ext_num = length([Particles_homo(i+1).ext_radius]);

elseif ll == 2

Particles_homo(i+1).peri_radius = [[Particles_homo(i).peri_radius], rcrit_homo(i,ll)*ones(dN_homo(i,ll),1)']; % add new nuclei of radius rcrit

Particles_homo(i+1).peri_num = length([Particles_homo(i+1).peri_radius]);

else

Particles_homo(i+1).cyto_radius = [[Particles_homo(i).cyto_radius], rcrit_homo(i,ll)*ones(dN_homo(i,ll),1)']; % add new nuclei of radius rcrit

Particles_homo(i+1).cyto_num = length([Particles_homo(i+1).cyto_radius]);

end

% == Update moles of available monomer

y(i+1,3,ll) = y(i+1,3,ll) - (dN_homo(i,ll)*vcrit_homo)/(v0*Na*vol(ll)); % gain in free monomer from chemistry - loss due to nucleation

% check if heterogenous nucleation can occur

if nucleation.enforce_number == 1

if ll == 1

nuc_sites(ll) = initial_nuc_sites(ll) - Particles_het(i).ext_num;

elseif ll == 2

nuc_sites(ll) = initial_nuc_sites(ll) - Particles_het(i).peri_num;

else

nuc_sites(ll) = initial_nuc_sites(ll) - Particles_het(i).cyto_num;

end

end

if nuc_sites(ll) > 0

%%%%%%%%%%%%

Rcap = 2*theta*gamma*v0/kT; %

rcrit_het(i,ll) = Rcap/log(S(i,ll)); % radius of new nuclei

% prevent non-physical nuclei forming

if rcrit_het(i,ll) <= r0

rcrit_het(i,ll) = r0;% if rcrit < radius of single atoms, force nuclei to be a single atom

end

vcrit_het = (4*pi/3)*rcrit_het(i,ll)^3; % volume of new nuclei

dG_het = 16*pi*(theta*gamma)^3*v0^2/(3*(kT)^2*log(S(i,ll))^2);

% NUCLEATION RATE (HETEROGENEOUS)

knuc_het = (y(i,3,ll)*Na*vol(ll))*(kT/(3*pi*(2*r0)^3*viscosity))*exp(-dG_het/kT);

dN_het(i,ll) = floor(h*(knuc_het));

% determine if there is enough CdS to nucleate all the particles

dN_het(i,ll) = min([ dN_het(i,ll), floor((y(i+1,3,ll)*Na*vol(ll))/(vcrit_het/v0)) ]);

% cap the maximum number of heterogenous NP formed

% if new batch of nuclei exceeds available nucleation sites, only add as many nuceli as available sites

if nucleation.enforce_number == 1

if dN_het(i,ll) > nuc_sites(ll)

dN_het(i,ll) = nuc_sites(ll);

end

end

else

nuc_sites(ll) = 0; % correct if negative

rcrit_het(i,ll) = 0;

vcrit_hetero = 0;

dN_het(i,ll) = 0;

end % end if nuc sites

% === update numbers of heterogeneously nucleated NP

if ll == 1

Particles_het(i+1).ext_radius = [[Particles_het(i).ext_radius], rcrit_het(i,ll)*ones(dN_het(i,ll),1)']; % add new nuclei of radius Rcrit (typically ~0.5nm (S=1000) - 1 nm (S=5))

Particles_het(i+1).ext_num = length([Particles_het(i+1).ext_radius]);

elseif ll == 2

Particles_het(i+1).peri_radius = [[Particles_het(i).peri_radius], rcrit_het(i,ll)*ones(dN_het(i,ll),1)']; % add new nuclei of radius Rcrit (typically ~0.5nm (S=1000) - 1 nm (S=5))

Particles_het(i+1).peri_num = length([Particles_het(i+1).peri_radius]);

else

Particles_het(i+1).cyto_radius = [[Particles_het(i).cyto_radius], rcrit_het(i,ll)*ones(dN_het(i,ll),1)']; % add new nuclei of radius Rcrit (typically ~0.5nm (S=1000) - 1 nm (S=5))

Particles_het(i+1).cyto_num = length([Particles_het(i+1).cyto_radius]);

end

% == Update moles of available monomer

y(i+1,3,ll) = y(i+1,3,ll) - (dN_het(i,ll)*vcrit_het)/(v0*Na*vol(ll)); % gain in free monomer from chemistry - loss due to nucleation

else % if S < 1 set nuc stuff to zero

rcrit_homo(i,ll) = 0;

rcrit_het(i,ll) = 0;

vcrit_homo = 0;

vcrit_het = 0;

dN_homo(i,ll) = 0;

dN_het(i,ll) = 0;

% if no new particles form, copy the previous array of particles

% to the next timepoint

if ll == 1

Particles_het(i+1).ext_radius = [[Particles_het(i).ext_radius], []]; % add new nuclei of radius rcrit (typically ~0.5nm (S=1000) - 1 nm (S=5))

Particles_het(i+1).ext_num = length([Particles_het(i+1).ext_radius]);

Particles_homo(i+1).ext_radius = [[Particles_homo(i).ext_radius], []]; % add new nuclei of radius rcrit

Particles_homo(i+1).ext_num = length([Particles_homo(i+1).ext_radius]);

elseif ll == 2

Particles_het(i+1).peri_radius = [[Particles_het(i).peri_radius], []]; % add new nuclei of radius Rcrit (typically ~0.5nm (S=1000) - 1 nm (S=5))

Particles_het(i+1).peri_num = length([Particles_het(i+1).peri_radius]);

Particles_homo(i+1).peri_radius = [[Particles_homo(i).peri_radius], []]; % add new nuclei of radius Rcrit

Particles_homo(i+1).peri_num = length([Particles_homo(i+1).peri_radius]);

else

Particles_het(i+1).cyto_radius = [[Particles_het(i).cyto_radius], []]; % add new nuclei of radius Rcrit (typically ~0.5nm (S=1000) - 1 nm (S=5))

Particles_het(i+1).cyto_num = length([Particles_het(i+1).cyto_radius]);

Particles_homo(i+1).cyto_radius = [[Particles_homo(i).cyto_radius], []]; % add new nuclei of radius Rcrit

Particles_homo(i+1).cyto_num = length([Particles_homo(i+1).cyto_radius]);

end

end % end of if S > 1

end % end of for ll = 1:3

%%%%%---------PARTICLE GROWTH----------%%%%%%%%%%

% grow particles that were homogeneously nucleated

% check if there are particles

if Particles_homo(i+1).ext_num>0

ll=1;

Rcap = 2*gamma*v0/kT;

Radius = Particles_homo(i+1).ext_radius;

monomer_to_growth = 0; % local variable to account for reduction in monomer conc due to growth

% go through and grow each particle

for g = 1:Particles_homo(i+1).ext_num

% ---- GROWTH RATE EQUATION -----

drdt(g) = (v0*Na)*C0_sat*D*(S(i,ll)-exp(Rcap./Radius(g))) / (Radius(g)+(D/kgr)*exp(alpha*Rcap/Radius(g))); % Eqn 14 from Talapin

Radius(g) = Radius(g) + h*drdt(g); % update radius

% Calulcate increase in volume to each NP

dVol = (4*pi/3)*((Radius(g))^3 - (Radius(g)-h*drdt(g))^3); % additional new volume per particle in m^3

% Calculate moles of monomer dedicated to growth

monomer_to_growth = monomer_to_growth + dVol/(v0*Na*vol(ll));

end

% update particle radii

Particles_homo(i+1).ext_radius = Radius;

% == Update moles of available monomer after growth

y(i+1,3,ll) = y(i+1,3,ll) - monomer_to_growth; % change in moles of free C0 due to growth + disolve

end

% next particles in periplasm

if Particles_homo(i+1).peri_num>0

ll=2;

Rcap = 2*gamma*v0/kT;

Radius = Particles_homo(i+1).peri_radius;

monomer_to_growth = 0;

% go through and grow each particle

for g = 1:Particles_homo(i+1).peri_num

drdt(g) = (v0*Na)*C0_sat*D*(S(i,ll)-exp(Rcap./Radius(g))) / (Radius(g)+(D/kgr)*exp(alpha*Rcap/Radius(g))); % Eqn 14 from Talapin

Radius(g) = Radius(g) + h*drdt(g); % update radius

% Calulcate increase in volume to each NP

dVol = (4*pi/3)*((Radius(g))^3 - (Radius(g)-h*drdt(g))^3); % additional new volume per particle in m^3

% Calculate moles of monomer dedicated to growth

monomer_to_growth = monomer_to_growth + dVol/(v0*Na*vol(ll));

end

Particles_homo(i+1).peri_radius = Radius;

% == Update moles of available monomer after growth

y(i+1,3,ll) = y(i+1,3,ll) - monomer_to_growth; % change in moles of free C0 due to growth + disolve

end

% next particles in cytoplasm

if Particles_homo(i+1).cyto_num>0

ll=3;

Rcap = 2*gamma*v0/kT;

Radius = Particles_homo(i+1).cyto_radius;

monomer_to_growth = 0;

% go through and grow each particle

for g = 1:Particles_homo(i+1).cyto_num

drdt(g) = (v0*Na)*C0_sat*D*(S(i,ll)-exp(Rcap./Radius(g))) / (Radius(g)+(D/kgr)*exp(alpha*Rcap/Radius(g))); % Eqn 14 from Talapin

Radius(g) = Radius(g) + h*drdt(g); % update radius

% Calulcate increase in volume to each NP

dVol = (4*pi/3)*((Radius(g))^3 - (Radius(g)-h*drdt(g))^3); % additional new volume per particle in m^3

% Calculate moles of monomer dedicated to growth

monomer_to_growth = monomer_to_growth + dVol/(v0*Na*vol(ll));

end

Particles_homo(i+1).cyto_radius = Radius;

% == Update moles of available monomer after growth

y(i+1,3,ll) = y(i+1,3,ll) - monomer_to_growth; % change in moles of free C0 due to growth + disolve

end

%%%%%%%%%%%%%%%%%%%%%%%

% grow particles that were heterogeneously nucleated

% check if there are particles

if Particles_het(i+1).ext_num>0

ll=1;

Rcap = 2*theta*gamma*v0/kT;

Radius = Particles_het(i+1).ext_radius;

monomer_to_growth = 0;

% go through and grow each particle

for g = 1:Particles_het(i+1).ext_num

% ---- GROWTH RATE EQUATION -----

drdt(g) = (v0*Na)*C0_sat*D*(S(i,ll)-exp(Rcap./Radius(g))) / (Radius(g)+(D/kgr)*exp(alpha*Rcap/Radius(g))); % Eqn 14 from Talapin

Radius(g) = Radius(g) + h*drdt(g); % update radius

% Calulcate increase in volume to each NP

dVol = (4*pi/3)*((Radius(g))^3 - (Radius(g)-h*drdt(g))^3); % additional new volume per particle in m^3

% Calculate moles of monomer dedicated to growth

monomer_to_growth = monomer_to_growth + dVol/(v0*Na*vol(ll)); % new

end

Particles_het(i+1).ext_radius = Radius;

% == Update moles of available monomer after growth

y(i+1,3,ll) = y(i+1,3,ll) - monomer_to_growth; % change in moles of free C0 due to growth + disolve

end

% next particles in periplasm

if Particles_het(i+1).peri_num>0

ll=2;

Rcap = 2*theta*gamma*v0/kT;

Radius = Particles_het(i+1).peri_radius;

monomer_to_growth = 0;

% go through and grow each particle

for g = 1:Particles_het(i+1).peri_num

drdt(g) = (v0*Na)*C0_sat*D*(S(i,ll)-exp(Rcap./Radius(g))) / (Radius(g)+(D/kgr)*exp(alpha*Rcap/Radius(g))); % Eqn 14 from Talapin

Radius(g) = Radius(g) + h*drdt(g); % update radius

% Calulcate increase in volume to each NP

dVol = (4*pi/3)*((Radius(g))^3 - (Radius(g)-h*drdt(g))^3); % additional new volume per particle in m^3

% Calculate moles of monomer dedicated to growth

monomer_to_growth = monomer_to_growth + dVol/(v0*Na*vol(ll));

end

Particles_het(i+1).peri_radius = Radius;

% == Update moles of available monomer after growth

y(i+1,3,ll) = y(i+1,3,ll) - monomer_to_growth; % change in moles of free C0 due to growth + disolve

end

% next particles in cytoplasm

if Particles_het(i+1).cyto_num>0

ll=3;

Rcap = 2*theta*gamma*v0/kT;

Radius = Particles_het(i+1).cyto_radius;

monomer_to_growth = 0;

% go through and grow each particle

for g = 1:Particles_het(i+1).cyto_num

drdt(g) = (v0*Na)*C0_sat*D*(S(i,ll)-exp(Rcap./Radius(g))) / (Radius(g)+(D/kgr)*exp(alpha*Rcap/Radius(g))); % Eqn 14 from Talapin

Radius(g) = Radius(g) + h*drdt(g); % update radius

% Calulcate increase in volume to each NP

dVol = (4*pi/3)*((Radius(g))^3 - (Radius(g)-h*drdt(g))^3); % additional new volume per particle in m^3

% Calculate moles of monomer dedicated to growth

monomer_to_growth = monomer_to_growth + dVol/(v0*Na*vol(ll));

end

Particles_het(i+1).cyto_radius = Radius;

% == Update moles of available monomer after growth

y(i+1,3,ll) = y(i+1,3,ll) - monomer_to_growth; % change in moles of free C0 due to growth + disolve

end

end

% make figures

figure(1);

subplot(3,1,1)

ax=gca;

plot(time(1:end-1),y((1:end-1),1,1)*1000,'Linewidth',2)

hold on

plot(time(1:end-1),y((1:end-1),1,2)*1000,'Linewidth',2)

hold on

plot(time(1:end-1),y((1:end-1),1,3)*1000,'Linewidth',2)

xlabel('time/hr');

ylabel('[Cd^{2+}]/mM')

xlim([0 1.05*max(time)])

legend('External','Periplasm','Cytoplasm')

legend('boxoff')

legend('location','best')

ax.FontName = 'Lucidia Sans';

ax.FontWeight = 'bold';

ax.FontSize = 12;

subplot(3,1,2)

ax=gca;

plot(time(1:end-1),y((1:end-1),2,1)*1000,'Linewidth',2)

hold on

plot(time(1:end-1),y((1:end-1),2,2)*1000,'Linewidth',2)

hold on

plot(time(1:end-1),y((1:end-1),2,3)*1000,'Linewidth',2)

xlabel('time/hr');

ylabel('[S^{2-}]/mM')

xlim([0 1.05*max(time)])

legend('External','Periplasm','Cytoplasm')

legend('boxoff')

legend('location','best')

ax.FontName = 'Lucidia Sans';

ax.FontWeight = 'bold';

ax.FontSize = 12;

subplot(3,1,3)

ax=gca;

plot(time(1:end-1),y((1:end-1),3,1)*1000,'Linewidth',2)

hold on

plot(time(1:end-1),y((1:end-1),3,2)*1000,'Linewidth',2)

hold on

plot(time(1:end-1),y((1:end-1),3,3)*1000,'Linewidth',2)

xlabel('time/hr');

ylabel('[CdS]/mM')

xlim([0 1.05*max(time)])

legend('External','Periplasm','Cytoplasm')

legend('boxoff')

ax.FontName = 'Lucidia Sans';

ax.FontWeight = 'bold';

ax.FontSize = 12;

figure(2)

vv = {'Extracellular Homo','Periplasm Homo','Cytoplasm Homo','Extracelluar Hetero','Periplasm Hetero','Cytoplasm Hetero'};

set(gcf,'Position', [1, 1, 700, 300]);

plot(time(1:end),[Particles_homo.ext_num],'Linewidth',2,'Color',c(1,:))

hold on

plot(time(1:end),[Particles_homo.peri_num],'Linewidth',2,'Color',c(2,:))

plot(time(1:end),[Particles_homo.cyto_num],'Linewidth',2,'Color',c(3,:))

plot(time(1:end),[Particles_het.ext_num],'Linewidth',2,'Color',c(4,:))

plot(time(1:end),[Particles_het.peri_num],'Linewidth',2,'Color',c(5,:))

plot(time(1:end),[Particles_het.cyto_num],'Linewidth',2,'Color',c(6,:))

ylabel('Number of Nanoparticles .')

xlabel('time/hr')

xlim([0 1.05*max(time)])

max_particles = max([[Particles_homo.ext_num], [Particles_homo.peri_num], [Particles_homo.cyto_num], [Particles_het.ext_num], [Particles_het.peri_num], [Particles_het.cyto_num]]);

if max_particles == 0

max_particles = 10;

end

ax=gca;

ax.YScale = 'log';

ax.YLim = [1e-0 max_particles*2];

ax.FontName = 'Lucidia Sans';

ax.FontWeight = 'bold';

ax.FontSize = 14;

pp = legend(vv{:});

pp.Box='off';

pp.FontSize = 10;

pp.FontWeight = 'normal';

pp.Location = 'best';
